# Artificial Intelligence Applications for Composite Materials: A Review

**DOI:** 10.3390/polym18192439

**Published:** 2026-10-07

**Authors:** Nada S. Alharthi, Laila M. Alqahtani, Albatool A. Abaalkhail, Ibtehal S. Baazeem, Mustafa Y. Haddad, Mohammed T. Alamoudi, Basheer A. Alshammari

**Affiliations:** 1 Advanced Materials Technologies Institute, King Abdelaziz City for Science and Technology, Riyadh 11442, Saudi Arabia; nalharthy@kacst.gov.sa (N.S.A.);; 2 Artificial Intelligence & Robotics Institute, King Abdelaziz City for Science and Technology, Riyadh 11442, Saudi Arabia

**Keywords:** artificial intelligence, machine learning, deep learning, generative AI, composite materials, damage detection, process optimization, physics-informed AI

## Abstract

Background: Composite materials are not easy to model using traditional experiments and physics-based approaches due to their diverse properties and multiscale nature. This review summarizes how artificial intelligence (AI) methodologies, including machine learning, deep learning, and generative AI, have been increasingly transforming and how they have been increasingly applied to composite materials research. It combines their possible use across the prediction of their properties, damage detection, structural health monitoring, and materials design. Methods: Searches were performed in Scopus and Google Scholar for articles published using combined keywords such as “(artificial intelligence OR machine learning OR deep learning OR Generative AI) AND (composite OR fiber reinforced) AND (prediction OR optimization OR characterization)”. The search strategy was designed to capture both fundamental developments in AI methodologies relevant to materials research and their specific applications to composite materials. The identified publications underwent a two-step selection process: (i) an initial review of titles and abstracts, and (ii) a detailed full-text evaluation. Studies were included if they provided explicit descriptions of AI models, clearly specified datasets, and applied quantitative performance assessments to composite materials-related applications such as material property prediction, damage detection, characterization, or manufacturing process optimization. For each selected article, relevant information was extracted in a structured and consistent manner, including the type of composite material, AI methodology, input parameters, dataset characteristics and size, validation methods, and application domain. These data were used to support both quantitative trend analysis and the qualitative identification of research gaps and future research opportunities in composite materials research. Results: Supervised learning methods, in particular artificial neural networks, support vector machines, random forests, and decision trees, were commonly used and exhibited durable potential accuracy in predicting properties of composite materials. Unsupervised methods such as principal component analysis (PCA) and clustering were comparatively underused. Generative AI, including generative adversarial networks (GANs) and variational autoencoders (VAEs), and physics-informed models emerged as growing approaches for synthetic data generation and improved interpretability, respectively. Limitations: The reviewed studies were frequently limited by small or heterogeneous datasets, weak generalizability testing beyond training conditions, high computational requirements and limited model interpretability (“black box” behavior); a risk-of-bias assessment across included studies was not formally conducted. Conclusions: AI is shifting composite materials research from trial-and-error toward data-driven, predictive design. Realizing its full potential will require open, well-annotated datasets, physics-aware and explainable models, and closed-loop, active-learning workflows linking prediction to experimental validation.

## 1. Introduction

The progress of the field of materials science and engineering continues to be motivated by the growing demand for materials resources with exceptional properties for progressive applications. Among these resources, composite materials offer outstanding combinations of properties and performance that exceed those of conventional majority materials. Composite materials are formed by combining two or more components with noticeably different chemical or/and physical properties, and they have significantly impacted industry sectors such as automotive, aerospace, energy, construction, oil and gas [1,2]. Due to their excellent weight-to-strength ratios, corrosion resistance, and design flexibility, these materials are recognized as preferred choices for many diverse applications [3,4].

Despite their transformative potential, the design, development, optimization, and fabrication of composite materials pose considerable challenges for scientists, researchers and engineers. The properties of such materials develop from complex interactions among their components at various length scales, ranging from the macroscopic structures of fibers or particles to the molecular interactions occurring at the interfaces [5,6]. For conventional analytical and experimental methods, their multiscale nature and the massive design space that includes reinforcement type and size, matrix material selection, orientation, processing parameters, and volume fraction of the reinforcement create a challenging environment [7].

Current developments in artificial intelligence (AI), including machine learning (ML), deep learning (DL), and generative AI (Gen AI), have significantly expanded the analytical and predictive capabilities available in composite materials research. These approaches enable the analysis of complex and high-dimensional datasets, allowing researchers to identify relationships between processing conditions, material structure, and resulting properties more effectively than with conventional approaches alone [8,9]. As a result, the field is gradually moving from mainly theory- and experiment-driven methods towards more data-informed strategies for material design and development.

In composite materials research, AI has shown strong potential in several key areas, including property prediction, microstructural characterization, damage detection, quality evaluation, process optimization, and materials design [10,11]. By supporting faster interpretation of large datasets and reducing dependence on extensive trial-and-error experimentation, these methods can help improve research efficiency, lower development time and cost, and support the design of higher-performance composite systems. Overall, the integration of AI into composite materials research is becoming an important step toward more efficient, accurate, and intelligent materials development.

Furthermore, the continuous integration of AI into composite materials research and development activities is expected to be shaped by advancements in data accessibility, modeling approaches, and computational frameworks. One of the key challenges remains data scarcity, particularly the limited availability of high-quality experimental datasets. To address this matter, multi-fidelity modeling approaches are gaining increasing attention, as they combine data from simulations, experiments, and literature sources to improve model robustness [12,13]. By leveraging abundant low-fidelity data alongside limited high-fidelity experimental data, these methods significantly enhance predictive accuracy while reducing experimental cost [14,15]. In addition, the integration of physics-informed machine learning and generative AI models is anticipated to further improve interpretability and enable inverse design of composite materials. This review intends to provide a comprehensive overview of the application of AI, particularly focusing on ML, DL and Gen AI, in the realm of composite materials. It will delve into the foundational concepts of both composites and AI, examining the various applications of different AI algorithms in the design, property prediction, optimization, and damage assessment of composite materials. Furthermore, the article discusses the implications of AI for the future of research in composite materials, highlighting significant benefits, current challenges, and promising future directions in this rapidly evolving interdisciplinary field. By synthesizing recent advancements, this paper aims to clarify the rationale behind the use of AI in composites and elucidate how it facilitates their synthesis and optimization. Eventually, it seeks to predict the future developmental path of composite materials through the application of AI.

To achieve this, we performed a comprehensive review to map the use of AI in composite materials research. Searches were performed in Scopus and Google Scholar for articles published using combined keywords such as “(artificial intelligence OR machine learning OR deep learning OR Generative AI) AND (composite OR fiber reinforced) AND (prediction OR optimization OR characterization)”. The search strategy was designed to capture both fundamental developments in AI methodologies relevant to materials research and their specific applications to composite materials. The identified publications underwent a two-step selection process: (i) an initial review of titles and abstracts and (ii) a detailed full-text evaluation. Studies were included if they provided explicit descriptions of AI models, clearly specified datasets, and applied quantitative performance assessments to composite materials-related applications such as material property prediction, damage detection, characterization, or manufacturing process optimization. Purely theoretical or conceptual studies, conference abstracts without full papers, non-English publications, and studies lacking experimental or numerical validation were excluded. For each selected article, relevant data were extracted in a structured and consistent manner, including the type of composite material, AI methodology, input parameters, dataset characteristics and size, validation methods, and application domain. These data were used to support both quantitative trend analysis and qualitative identification of research gaps and future research opportunities in the composite materials area.

To move beyond a method-by-method overview, this review adds a synthesis layer that guides the researcher in selecting optimal approaches, validating results, and identifying relevant research gaps. Overall, the manuscript cites 172 references, encompassing original research articles, relevant review papers, and methodological and background studies. The literature is organized using a task-driven view including property prediction, damage or structural health monitoring (SHM), process optimization, and design, while also linking each task to the most common data modalities and learning types used in practice. We then identify common technical limitations across the reviewed studies and discuss practical considerations for model development and evaluation. Finally, we outline future research directions, with particular emphasis on physics-aware modeling, data-efficient learning, active learning, and digital twins as potential approaches for improving the reliability and practical use of AI in composite material engineering.

To provide a quantitative overview of the literature included in the analysis, the eligible AI-based composite studies were further classified according to the principal AI methodology and composite material group. Table 1 summarizes the resulting distributions using two complementary measures: the proportion within the eligible studies included in the quantitative analysis and the corresponding proportion relative to the total references cited in the manuscript. Traditional machine learning approaches represent the largest AI category, followed by artificial neural networks and deep-learning methods. In terms of material systems, general or heterogeneous composites constitute the largest group, followed by polymer/hybrid composites and fiber-reinforced polymer composites.

Several reviews have already examined the intersection of AI and composite materials, but each has generally been confined to a single AI paradigm, a single composite class, or a single application. For example, Agarwal et al. [12] focused on multiscale computational and AI models for linear and nonlinear composites, Ribeiro Junior and Gomes [16] restricted their scope to machine learning for damage assessment, Karuppusamy et al. [17] and Kordijazi et al. [18] surveyed machine learning applications limited to polymer composites and metal matrix composites, respectively, and Sorour et al. [19] focused on predicting and optimizing the mechanical behavior of laminated fiber-reinforced polymer composites, while Daghigh et al. [20] reviewed machine learning applications for defect detection in composite materials. Additionally, Hrehova et al. [21] studied applied ML to predict the absorption properties of recycled polyvinyl butyral-based composite materials obtained from automotive windshields. The ML approach was used to analyze the influence of material composition and predict composite behavior.

In contrast, the present review integrates supervised, unsupervised, reinforcement, and generative AI approaches across multiple composite material class research tasks, property prediction, damage and structural health monitoring, process optimization, and materials design. Beyond this broader scope, the novelty of this work lies in its quantitative analysis of the literature covered by the 172 references included in this review, enabling trend analysis across AI methodology, application domain, and composite type; its identification of repeated technical bottlenecks concentrated into practical recommendations for model development and validation; and its forward-looking roadmap linking physics-informed modeling, active learning, and digital twin concepts to reliable engineering deployment, an integrative perspective that, to our knowledge, has not been previously presented in a single previous review.

## 2. Background

### 2.1. Composite Materials

Composite materials are engineered materials made from two or more constituents with noticeably different chemical or physical properties. When combined, these materials yield a final product that differs from the original components [16,17,18,19,20,21,22]. The individual components remain distinct within the new material’s structure, which differentiates composites from mixtures and solid solutions [23]. The main goal of combining these base materials is to develop a new material that displays enhanced qualities or a synergistic blend of properties that cannot be achieved by the individual components alone [24]. The usage of composite materials has a long history, but their up-to-date applications have meaningfully expanded in recent years due to their valuable properties [25]. The main structure of a composite material characteristically consists of two main components: a matrix material and a reinforcing phase. The matrix, which can be made of polymeric, metallic, or ceramic ingredients, serves numerous purposes. For example, it binds the reinforcement, transfers load between them and protects them from environmental damage and external forces. The reinforcing phase, usually in the form of fibers, particles, or whiskers, adds strength, rigidity, and stiffness to the composite in general. They also help to prevent crack propagation and fractures within the material. The combination of the matrix and the reinforcing materials gives composites their excellent tensile strength, flexural strength, thermal properties, and resistance to environmental degradation [1,12,22,26].

Composite materials can be divided into several main types based on their matrix and reinforcement compositions. Polymer matrix composites represent the most widespread class, distinguished by their high strength-to-weight ratio, exceptional corrosion resistance, recyclability and versatility in being shaped or designed according to target applications. Typically, a polymer resin, such as a thermosetting resin, includes an epoxy, a polyester, a vinyl ester, or a thermoplastic like polyethylene and polystyrene, serves as the matrix and is reinforced with fibers like carbon, glass, or aramid. Due to these beneficial and advantageous properties, polymer matrix composites are widely employed in industry applications [27,28].

Metal matrix composites encompass metal and even alloy matrices reinforced with particles, whiskers, or fibers. This configuration allows for improved mechanical properties, enhanced wear resistance, and increased thermal stability, rendering metal matrix composites suitable for demanding applications requiring superior performance and durability. Outstanding examples include aluminum matrix composites reinforced with silicon carbide or alumina for aerospace structural parts [29,30]. Ceramic matrix composites contain a ceramic matrix reinforced with particles, whiskers, or fibers, such as silicon carbide fibers or alumina for reentry vehicles. Their outstanding thermal stability, remarkable resistance to oxidation, corrosion, and ablation, as well as minimal creep, make them particularly advantageous for high-temperature applications. Consequently, ceramic matrix composites are essential for lightweight structural and thermal-management applications such as components in aircraft engines and gas turbines [31].

Natural fiber composites (NFCs) utilize natural fibers, such as jute, hemp, bamboo, or wood, as reinforcement within a polymer matrix. These composites offer environmentally friendly and biodegradable alternatives, delivering cost-effective and lightweight solutions. Their sustainability and biodegradability have attracted considerable research interest, encouraging the adoption of natural fibers and decreasing dependency on synthetic reinforcements [32]. Lastly, hybrid composites combine two or more types of base materials or different forms of reinforcement, frequently at the nanometer or molecular level, to optimize material properties. By integrating tailored structural characteristics with functionalities such as energy storage and sensing, they enable the development of advanced smart material systems [33]. Figure 1 shows an overview of different types of composite materials that can be utilized in predicting their properties, detecting damage, and accelerating the development of their composite systems using AI.

### 2.2. Artificial Intelligence in Materials Science

The term AI was first introduced in 1955 by John McCarthy and gained prominence during the Dartmouth Summer Research Project on Artificial Intelligence held in 1956 at Dartmouth College. At that time, early algorithms had already enabled computers to solve algebraic problems and process basic English sentences [34,35]. Since its beginning, AI has continued from a principally academic effort into a foundational element of widely adopted technologies, for example, medical diagnostics, speech recognition, autonomous transportation, and intelligent virtual assistants [26,27]. This transformation has been driven by sustained advances in computational capabilities and the development of more sophisticated algorithms, enabling the transition of AI from theoretical models to practical real-world applications.

AI is a broad discipline that aims to develop computational systems capable of mimicking human cognitive functions by processing data from diverse sources, learning from patterns, and performing tasks typically associated with human intelligence, such as decision-making, problem-solving, and language comprehension [36]. These capabilities have substantially impacted materials science, facilitating a shift from traditional trial-and-error experimentation to a data-driven paradigm characterized by design and predictive modeling, thereby enabling more efficient improvements and discoveries.

AI has demonstrated remarkable effectiveness in addressing a wide range of contemporary challenges. It is highly efficient in solving hard problems that are often beyond the capabilities of conventional computational approaches. The incorporation of AI into the design and production of composite materials has introduced novel methodologies to drive progress in the field [37]. By transforming data analysis and computational modeling, AI techniques have contributed to increased efficiency [17], accelerated design cycles [38], the development of high-performance composite materials [39], reduced material waste [27], and improved system reliability [40]. However, despite its considerable potential in composite material engineering, the application of AI remains constrained by several challenges, such as poor data quality, low interpretability of computational models, and broader ethical issues [17].

To gain a clear understanding of how AI drives and enables these technologies, it is important to outline the key AI subfields used and applied in composite materials research [41]. ML is a subfield of AI that focuses on learning and identifying patterns within data to generate predictions or support decisions [42]. DL is an advancement of ML that leverages multilayered neural networks, known as deep neural networks, to analyze complex data and improve performance in tasks such as image recognition and natural language processing [43]. Gen AI is a branch of AI that creates novel content by learning patterns from existing data and is primarily enabled by DL models [44].

#### 2.2.1. Machine Learning (ML)

ML, a branch of AI, involves developing algorithms that can automatically learn patterns and relationships directly from data, rather than relying on explicitly programmed rules for the targeted task [45]. They learn by inferring relationships between inputs and outputs in the training phase and can generalize to new, unseen data, which can be used for broader, similar tasks [46]. The knowledge gained from training ML models can be analogous to scientists building intuition over many years of acquired research experience. These models provide powerful tools for predicting the behavior, performance and properties of new materials, facilitating the design and development of novel composite materials with tailored performance characteristics [47].

Composite material design involves a wide range of design parameters and requires a large design space. In addition, they typically exhibit more anisotropic mechanical properties than homogeneous or isotropic materials. Owing to these characteristics, they necessitate the use of larger and more complex datasets, rendering their design and optimization processes markedly more challenging. Therefore, a critical challenge for the scientific community is the effective integration of AI methodologies to accelerate the design, iterative optimization, and high-level engineering applications of composite materials [38]. In composite materials research, ML addresses three primary challenges [39]:

1. Property Prediction: Developing quantitative structure and property relationships using compositional, process-specific, and structural data.
2. Process Optimization: Leveraging experimental or simulation data to optimize manufacturing parameters.
3. Quality Control: Detecting anomalies and errors in production data.

For composite materials, the ML workflow includes three critical steps: (1) data preparation, which involves the preprocessing of the curated materials data to resolve issues such as missing data, noise, and outliers; (2) model training, which aims to identify relationships between design parameters and properties; and (3) model evaluation, which assesses the ability of the model to generalize using appropriate validation methods, as shown in Figure 2.

Typically, raw data are generated using numerical simulations [48] and experimental procedures [49]. However, during the data acquisition process, these data often suffer from some quality issues, including missing or incomplete information [50], high levels of noise [51], discrepancies, and outliers [50]. Therefore, data preprocessing techniques can effectively enhance data quality and reliability. For instance, data imputation can efficiently address missing values and enhance the completeness of datasets. Noisy data can be minimized using techniques such as binning or clustering [52]. Moreover, feature importance analysis assists in identifying the input variables that drive model predictions the most, reducing complexity and improving prediction accuracy. For instance, more than ten design parameters are required to predict the buckling load of composite materials. However, only four factors are most relevant: the ratio of tensile stiffness to shear stiffness, the ratio of the tensile stiffness of the plate to that of the rib, the orthotropic ratio of the stiffened plate, and the shear buckling parameter [40]. Therefore, appropriate preprocessing techniques should be selected carefully during data preparation to ensure the development of accurate, reliable, and fast-converging models.

AI models are typically trained once the input and output variables are defined and suitable datasets are prepared [53]. Among the various AI modeling approaches, neural networks have emerged as one of the most widely used architectures. These networks operate as “black box” systems wherein relationships between neurons across multiple layers are established through linear or nonlinear functions, enabling the mapping of input features to predicted outputs [41]. In composite materials, it remains challenging to model the complex and often nonlinear relationships between design parameters and materials performance using conventional theoretical approaches. AI-based methods overcome this limitation by learning directly from data, allowing for more effective and accurate mapping, prediction and generalization of relationships between parameters and the performance of the developed composite materials [42,43]. For example, in two-phase or multi-phase composites, it is difficult to characterize the influence of microstructural topologies on mechanical behavior owing to the lack of analytical models that account for the effects of varying distributions of reinforcement phases within the matrix.

The performance of an AI model should be assessed once its training is completed. Model evaluation determines the accuracy and generalizability of the model when applied to unseen data, thereby guiding the selection of an AI model that offers reliable predictions and robust performance [46]. To simplify this process, the dataset is typically divided into three subsets: a training set, used for model learning, a validation set, used for tuning the model’s hyperparameters, and a test set, used for model evaluation [54]. Commonly used dataset division techniques include the holdout method [47], K-fold cross-validation method [55], and bootstrap method [56]. A single holdout split can give a misleading estimate of performance when datasets are small or class-imbalanced, as is often the case in composite materials research. Stratified k-fold cross-validation reduces this variance by averaging results over several held-out folds. Nested cross-validation goes further by separating hyperparameter selection from the final performance estimate, which avoids the optimistic bias that arises when the same data are used both to choose a model and to evaluate it [57]. External validation is equally valuable: applying the trained model to data from independently sourced material systems or processing conditions not represented in training tests whether it has captured transferable structure–property relationships or merely the peculiarities of one dataset [58]. Once an evaluation strategy has been chosen, model predictions are compared against the measured values in the test set. The appropriate performance metric depends on the task, such as accuracy [48], precision [49], recall, and the F1 score as the harmonic mean of precision and recall [50]. For regression tasks, performance is typically evaluated using the coefficient of determination (R^2^), mean absolute error, and mean squared error, alongside root mean squared error (RMSE) and mean absolute percentage error (MAPE) [21].

Recent studies demonstrated that ML algorithms increased prediction accuracy and expedited research on reinforced polymer composites and hybrid material systems [51]. In addition, it has also been successfully applied across several domains of materials science, such as additive manufacturing, where it plays a critical role in quality control, process optimization, and modeling of complex systems. Extensive ongoing research efforts aim to further refine ML models and expand their applicability, with the potential to reshape materials science into a more data-driven, innovative, and predictive discipline [52]. In addition to the issue of generalizability and model performance, unexamined variations in predicted or target values can lead to unreliable and irreproducible predictions. These uncertainties can be either data-inherent from the intrinsic randomness and noise within the data or stemming from the model’s limitations in understanding the data distribution. Often, in real applications, both types can appear when implementing AI models. As a result, incorporating uncertainty quantification (UQ) methods becomes crucial to validating any worthy predictions. Bayesian-based methods such as Gaussian process regression (GPR)and Bayesian neural networks (BNN) incorporate prior distributions to handle uncertainty, whereas sampling methods like Monte Carlo (MC) dropout approximate complex posterior distributions through stochastic sampling. Moreover, aspects like calibration, sharpness, reliability, and practical utility across different tasks provide a closer lens on the uncertainty performance of any related AI applications [15,58].

#### 2.2.2. Deep Learning (DL)

DL is a specialized branch of ML that utilizes multilayered artificial neural networks (ANNs) to analyze complex data and support decision-making. These networks are inspired by the structure and function of the human brain, which is composed of interconnected computational nodes (or “neurons”) that collectively work to identify complex patterns in data [59]. Unlike conventional ML approaches, which often rely on manually engineered feature extraction, DL models can automatically learn to extract hierarchical representations from raw and/or unstructured data. This capability enables them to effectively address a wide range of applications and tasks such as speech recognition, image processing, understanding natural language, and even content generation. While ANN architectures are inspired by the human brain, it is important to recognize that these models are simplified abstractions and do not fully replicate the complexity of biological neural processes [45,60].

In recent years, DL techniques have been increasingly applied to the discovery and analysis of new materials [61], with particular emphasis on properties such as plasticity, elasticity, fatigue life, buckling behaviors, and wear resistance [62]. Although these developments highlight the potential of DL in materials science, most existing efforts remain predominantly focused on predicting structural or material properties [63], rather than on the inverse designing of new materials tailored to exhibit specific characteristics.

Although numerical and analytical tools such as molecular dynamics simulations and finite element analysis (FEA) can support the composite design process, their high precision often entails substantial time and computational cost. Fortunately, the growing availability of experimental data, simulation outputs, and research repositories has enabled DL models to play a greater role in data-driven composite design, potentially improving material performance predictions and reducing overall design time. The accurate selection of the appropriate neural network based on the material’s data structure is critical to building effective DL models, as shown in Figure 3. Typical data structures in materials science can range from scalar descriptors to complex spatial or temporal structures such as vectors, images, graphs, and time series [41].

Composite materials are engineered to meet specific mechanical and environmental requirements, but their design involves numerous interacting variables related to material selection, fabrication processes, and structural configuration. Generating comprehensive datasets remains challenging because these variables strongly influence composite performance. Consequently, conventional design often requires extensive iterative optimization to achieve targeted multifunctional properties while minimizing weight. Moreover, vector-based data can represent material, processing, and design parameters. DL models, including fully connected neural networks and autoencoders, can use these data to learn complex structure–property relationships and support property prediction and design optimization [64,65,66].

Image-based data can be broadly categorized into two-dimensional (2D) and three-dimensional (3D) images, which are difficult to parameterize using discrete variables. High-resolution imaging data obtained from scanning electron microscopy (SEM) and transmission electron microscopy (TEM) provide spatially resolved information about materials, whereas X-ray diffraction XRD patterns provide structural and phase-related information. These data are commonly processed by extracting spatial or pattern-based features using a series of connected convolutional layers, which collectively form a convolutional neural network (CNN). CNNs can rapidly analyze image data, detect material defects [65], and interpret XRD patterns [66], enabling the identification of material phases and compositions [67]. Furthermore, CNNs may be integrated with acoustic emission (AE) or fully connected neural networks for tasks such as dimensionality reduction and optimization [68].

Time-dependent phenomena such as stress–strain responses, cyclic fatigue behavior, and electromagnetic signal propagation require models capable of capturing sequential dependencies. Recurrent neural networks are particularly well suited for modeling such applications because they feed the output back into the input layer and incorporate information from previous time steps into the current computation effectively, which maintains a memory of the previous states. However, for history-dependent and time-sensitive material behavior, advanced sequence models such as long short-term memory (LSTM) networks, gated recurrent units, and temporal convolutional networks can capture long-term dependencies and irreversible responses more effectively than conventional recurrent architectures [69,70,71,72]. For complex cyclic loading, time-aware recurrent models can further improve the representation of path-dependent behavior and maintain stable gradients [73]. More recently, physics-informed Transformers have shown potential for capturing long-range dependencies while incorporating physical constraints, enabling relationships between material descriptors and properties to be modeled more effectively [73,74].

Graph-based representations are particularly suitable for composite materials because they can capture interactions among constituents, interfaces, particles, and microstructural features. Graph neural networks (GNNs), including graph attention and graph convolutional networks, can exploit these relationships to predict material properties and characterize complex composite architectures [75,76,77,78]. The effectiveness of these models depends strongly on aligning the representation of the composite microstructure with the selected network architecture [79,80,81].

## 3. AI Algorithms in Composite Materials

AI methods employed in composite engineering can be categorized based on the learning model used to map data into predictions or decisions. This section first discusses supervised and unsupervised learning, followed by a brief review of reinforcement learning (RL) as distinct families, which are being increasingly reported in the composite materials literature. Across all models, two choices drive performance and credibility: (i) how the composite system is represented, that is, whether through tabular data, images, or graph-structured data capturing plies, interfaces, or microstructural topology; and (ii) how models are validated to avoid overly optimistic estimates, that is, whether appropriate test splits, nested cross-validation, calibrated probabilities, or class imbalance adjustments (particularly for defect detection) are employed [57]. Given that composite materials datasets are often small and noisy, robust baselines such as linear models, support vector machines (SVMs), Gaussian processes, and tree ensembles remain essential tools for ensuring reliable results [82,83].

As discussed earlier in Section 2.2.1, several ML models have been developed and applied in the field of composite materials, contributing to significant advances in material characterization, failure prediction, and manufacturing process control. Supervised learning models, such as ANNs, SVMs, and random forests (RFs), are typically used for tasks such as mechanical and property prediction and defect classification [84]. Unsupervised learning models, such as principal component analysis (PCA) and clustering, are typically used to reduce the dimensionality of the data and uncover hidden patterns within high-dimensional experimental datasets [85]. RL methods, such as deep RL (DRL), can be applied to the optimization process of digital materials, enabling the efficient navigation of vast design spaces to achieve targeted physical properties [86]. Generative approaches, including variational autoencoders (VAEs) and generative adversarial networks (GANs), have demonstrated strong potential and effectiveness in producing new candidates that satisfy the specified performance criteria [87].

### 3.1. Supervised Learning

Supervised learning is a machine learning paradigm in which models are trained using labeled datasets, where each input relates to an identified output or target value. During the training process, the model learns the underlying relationship between the input and the output features and subsequently uses this knowledge to predict outcomes for previously unseen data [88]. Supervised learning tasks are commonly divided into two main categories: classification and regression. Classification models assign inputs to predefined categories, and common classification techniques include logistic regression, decision trees (DTs), SVMs, k-nearest neighbors, and naïve Bayes classifiers. In contrast, regression models predict continuous numerical values. Regression analysis encompasses a family of approaches used to model the relationship between a dependent variable and one or more independent variables. Common regression techniques include linear regression (LR), polynomial regression (PR), support vector regression (SVR), principal component regression (PCR), and regularized methods such as ridge, lasso, and elastic net regression.

In the context of composite materials research, supervised learning algorithms map diverse data representations such as composite forms, non-destructive testing signals, and images to label parameters, such as strength, defect class, porosity, and bond strength. For structured tabular data, models such as SVMs, RFs, and DTs generally perform well, particularly when the variable relationships are linear. For high-dimensional inputs such as signals and images, ANNs are better suited, provided the training and test data are representative of the same underlying distribution [45]. Several supervised learning methods are discussed in greater detail in the following subsections, along with their applications in composite material engineering.

#### 3.1.1. Support Vector Machines (SVMs)

SVMs, a class of supervised ML algorithms, are being increasingly applied in the field of composite materials research. SVMs are particularly effective for addressing classification and regression problems in materials science owing to their ability to handle high-dimensional nonlinear datasets with limited samples [89].

Wang et al. [90] presented a novel image-based framework that integrated SVM-based classification with the Interface-Modified Reproducing Kernel Particle Method to model heterogeneous materials using micro-CT images. In this framework, the SVM generated signed distance scores, which guided the placement of interface nodes and the adjustment of kernel functions, thereby enabling the model to obtain accurate reproducing kernel particle method (RKPM) approximations across weak discontinuities. This framework enabled effective identification of material phases and material interfaces and was seamlessly incorporated with smoothed-cell nodal integration in 3D. The method was validated using both synthetic and SEM-derived 2D and 3D images, demonstrating its ability to accurately capture complex microstructures, making it effective for 3D simulations based on stacked image slices. Although binary SVMs were employed for two-phase segmentation, the framework is adaptable to more advanced ML models, such as multi-class SVMs and CNNs, to address complex multi-phase materials.

When it comes to property prediction, Chen [91] investigated the use of SVMs and ensemble learning to predict the power conversion efficiency (PCE) of semiconductor polymers for organic photovoltaic (OPV) devices, solely relying on the chemical structures of the polymers. Using Morgan fingerprints generated from SMILES codes and a dataset of 1203 polymers with their corresponding material and device properties, the SVM model achieved a correlation coefficient (CC) of 0.63, which was slightly outperformed by an RF model with a CC of 0.64. The prediction accuracy improved further when both models were combined through ensemble learning, reaching a correlation of 0.653. This approach highlights the potential for high-throughput virtual screening of OPV materials using ML. However, the study reported that the variability of experimental PCE measurements limits predictive accuracy, and higher-quality data could enhance model performance. Overall, the work demonstrated how AI can accelerate material discovery in organic electronics.

Elajjani et al. [92] developed a data-driven SVR model to estimate flow stress as a function of temperature, strain, and strain rate. Similarly, Owolabi et al. [93] developed two predictive models, particle swarm optimization (PSO)-based SVR (PSVR) and ordinary LR, to estimate the refractive index (*n*) and energy gap (E) of polyvinyl alcohol composite materials. The PSVR models (n-PSVR and E-PSVR) significantly outperformed the ordinary LR counterparts in terms of RMSE and MAPE, demonstrating higher predictive accuracy. These models were used to analyze the effects of sodium-based dysprosium oxide and benzoxazine derivatives on the energy gap of the composite and exhibited a strong agreement with the experimental data. External validation further confirmed their reliability with low mean absolute percentage errors. By optimizing model hyperparameters using PSO, the approach ensured high precision and robustness. Collectively, this modeling framework offers a cost-effective alternative to experimental trials for optimizing the optical properties of polyvinyl alcohol composites.

In a comprehensive comparative study, Bhattacharya, S. et al. [43] investigated the effectiveness of nine different regression models, namely LR, PR, SVR, PCR, quantile regression, median regression, ridge regression, lasso regression, and elastic net regression, as predictive tools for estimating response values during the turning and drilling of composite materials. The SVR model consistently outperformed the other models in both machining processes, achieving the lowest values for MAPE, root mean squared percentage error, root mean squared logarithmic error, and relative root squared error, with LR and PR following closely. By contrast, ridge and median regression models demonstrated the weakest predictive performance. These findings were further supported by Friedman rank and aligned rank tests. The durable performance of the SVR model was ascribed to its fewer tuning parameters, robustness, and ability to model both linear and nonlinear relationships. Additionally, the Wilcoxon test revealed statistically significant differences in prediction accuracy between certain models, specifically between LR and PCR, as well as between PR and quantile regression, for the drilling process at a 5% significance level. Despite relying on small datasets, this study highlights the potential of these models for machining applications.

#### 3.1.2. Decision Tree (DT)

The DT method was first introduced by D. Steinberg [83] in his seminal work Classification and Regression Trees (CART), where it was recognized for its clarity and effectiveness in decision-making. Since then, it has gained widespread adoption across various fields. A DT operates by hierarchically partitioning input data in a treelike structure. Its principal goal is to develop a model that can predict the values of target variables using multiple input features. When the target variable is continuous, the model is referred to as a regression tree; when the target variable is categorical or discrete, it is known as a classification tree.

Recent studies have explored tree-based algorithms for material selection and process optimization in engineering and manufacturing applications. For example, in fused deposition modeling of polyethylene terephthalate glycol, Barrios and Romero compared random forests (RF) and random tree (RT) models for predicting surface roughness using process variables such as layer height, extrusion temperature, print speed, print acceleration, and flow rate. In their study, the RT model showed the best predictive performance for that specific dataset. However, given the limited dataset size and the model-specific implementation used, this result should be interpreted as dataset-specific rather than as evidence of a general advantage of RT over RF [94].

Primary structures in launch vehicles, such as cylindrical shells, are increasingly being constructed using monolithic and sandwich composite materials. These structures are prone to buckling under axial compression caused by the weight of upper stage components and are very sensitive to imperfections. In composite material shells, both the buckling load and sensitivity to imperfections are influenced by the laminate stacking sequence. Fawad et al. [95] conducted multi-objective optimizations to determine the optimal stacking sequences for composite cylinders subjected to axial compression, considering various geometric imperfection types and employing a brute force approach using the DT model. In their study, DT-based ML was utilized to extract general design guidelines that maximize buckling resistance while minimizing sensitivity to imperfections. These policies were derived based on comparative stiffness properties, including Young’s modulus, tensile strength, flexural modulus, bending, in-plane shear, and torsional stiffness of graphene nanoplatelet-reinforced cementitious composites. The study produced several optimal stacking configurations, which were compared with existing designs from the literature. The DT model achieved a correlation coefficient (R) of 0.8708, demonstrating its ability to capture relationships between material parameters and compressive strength. Thus, the findings indicate that the proposed design recommendations result in high-performance cylinders that substantially outperform comparable composite shells. These insights could serve as a foundation for future lightweight designs of monolithic and sandwich composite structures in next-generation launch vehicles.

Materials used in extreme high-temperature environments, such as aircraft engines, gas turbines, nuclear reactors, reentry vehicles, and hypersonic structures, must withstand severe thermal and mechanical stresses. CMCs are well suited for these applications because they have high melting points, are highly resistant to oxidation, corrosion, and ablation, have low creep rates, and perform well under thermal cycling. The addition of boron nitride nanoparticles and continuous fibers renders them even more heat-resistant, stronger, and more resistant to breaking. Deb et al. [96] designed and tested six ML regression models to predict the behavior of these advanced composites during ablation. These models included DT, RF, SVM, gradient boosting, extreme gradient boosting, and adaptive boosting. Gradient boosting and extreme gradient boosting yielded the most accurate predictions. Their research shows that ML can be a valuable and dependable way to model the behavior of high-performance CMCs in extreme conditions.

#### 3.1.3. Random Forests (RF)

RF is an ensemble learning method that creates multiple DTs and combines their output predictions to improve prediction accuracy while reducing the risk of overfitting. As illustrated in Figure 4, it relies on the collective predictions of numerous DTs, improving forecast reliability through averaging and majority voting [97].

The versatility of RF across different composite material tasks is well documented in the literature. In the context of damage characterization, Wang et al. [99] employed RF to classify AE signals across various cutting speeds and fiber orientations, exploiting the time-frequency characteristics of the signals as discriminative features. For structural performance prediction, Zhang and Xue [100] applied the ensemble RF technique to predict the bond strength of near-surface-mounted fiber-reinforced polymer rods, demonstrating that the RF method outperforms the Gene Expression Programming (GEP) approach for this task.

Xiang Gao et al. [101] introduced an ML approach to predict porosity in carbon fiber-reinforced CMCs, supporting design efforts in material genetic engineering. Additionally, RF regression (RFR) and SVR models were developed using 1674 experimental records and nine input variables. The RFR model outperformed the SVR model, achieving higher accuracy and demonstrating its effectiveness in predicting porosity for material performance optimization.

Additionally, RF has also shown strong predictive capability for the mechanical properties of fiber-reinforced concrete. Almohammed et al. [57] employed RF and RT models to predict the splitting tensile strength of basalt fiber-reinforced concrete (BFRC). The dataset was compiled from various publications, each featuring different BFRC content, fiber lengths, and additional input parameters. Both RF and RT models were used to estimate the splitting tensile strength, with the RF model demonstrating superior performance in a comparative evaluation. The RF model achieved a higher correlation coefficient (0.9889 vs. 0.9579) and lower error metrics (MAPE: 0.0886 vs. 0.1842; RMSE: 0.1430 vs. 0.2406) than the RT model. Sensitivity analysis further revealed that curing time was the most critical determinant of splitting tensile strength.

Consistent with these findings, a comparative study [102] compared RF to DT, ANNs, and GEP models to predict the compressive strength of high-strength concrete. Using features such as cement content, aggregate ratios, water content, and superplasticizer dosage, the RF model achieved the highest predictive accuracy (R^2^ = 0.96) by leveraging DTs as ensemble learners. Additionally, both DT and GEP models exhibited strong predictive capability (both R^2^ ≈ 0.90) compared to ANNs (R^2^ ≈ 0.89).

Moreover, RF has been applied to assess long-term material durability. A study [103] developed an RF regression model for predicting the tensile-strength retention of laboratory-conditioned bars in an alkaline environment, using a comprehensive database of 772 tested specimens. The model was further employed for sensitivity and parametric analyses, as well as for evaluating the long-term performance of glass fiber-reinforced polymer rebars in alkaline concrete conditions. Collectively, these studies demonstrate that RF consistently exhibits strong predictive performance across diverse composite material applications with low error metrics across varied datasets and materials.

#### 3.1.4. Artificial Neural Networks (ANNs)

ANNs are data-driven models inspired by the structure and functioning of the human brain. They are particularly effective in handling multivariate and nonlinear relationships between input and output data. An ANN contains multiple interconnected layers of nodes (neurons), and it learns using training algorithms such as backpropagation and stochastic gradient descent [104]. In composite materials research, ANNs are widely applied for several applications, such as predicting the mechanical properties of composites, detecting damage, optimizing fabrication processes, and modeling material behavior under different loading conditions. Therefore, ANNs are mostly effective when the relationship between composition, processing parameters, and composite material properties is vastly nonlinear. However, their performance is strongly dependent on dataset size and quality, and they commonly provide lower interpretability than DT-based models. In contrast, RF models can remain competitive with smaller datasets and provide feature-significant information, making them attractive for primary stage composite development.

Dashtgoli et al. [105] compared three ML models: generalized regression neural network (GRNN), extreme learning machine (ELM), and SVR to predict the mechanical behavior of bio composite structures subjected to quasi-static out-of-plane loading. Among these, the GRNN model provided the most accurate predictions of nonlinear load–displacement behavior. Extending the application of ANNs to mechanical responses, Malashin et al. [106] investigated the use of an ANN for predicting various mechanical properties of textile composites, including static and dynamic behavior, time-dependent responses (such as creep and stress relaxation), fatigue life, wear performance, and crack detection. The study highlighted recent advancements in the ANN-based modeling of fiber-reinforced composites and underscored the importance of accurate property prediction for engineering and design applications.

Focusing precisely on wear behavior, Kumar et al. [107] employed a backpropagation-trained ANN to predict wear-induced height loss in aluminum alloy and their composite materials reinforced with aluminum oxide particles. Experimental data for model development were obtained from pin-on-disk tests conducted in accordance with ASTM G99, following a Taguchi L27 orthogonal design. Density, applied load, reinforcement weight fraction, and sliding distance served as input variables, enabling the ANN to model the nonlinear relationship with wear-induced height loss. The model predictions agreed well with experimental measurements, demonstrating their ability to capture the wear response across the investigated conditions. Among the tested compositions, the composite containing 6 wt.% aluminum oxide exhibited the highest wear resistance. Overall, the well-trained ANN proved an effective tool for accurately predicting wear performance in metal-based composite materials.

ANNs have also been applied to microstructure-property relationships. For example, Joshua et al. [108] utilized CNNs as a specialized type of ANN that excels in processing grid-like data, particularly images and videos, to model the relationship between ceramic microstructures and their ionic conductivity. The CNNs were trained using supervised learning on cropped microscope scan images. The corresponding macroscopic ionic conductivities were obtained through impedance spectroscopy and were used as labels. In another study, Cang et al. [109] proposed a morphology-aware generative model based on a VAE to generate stochastic microstructure samples. The generated samples were used as augmented training data to improve the performance of a CNN model for predicting material properties from sandstone microstructures. Alongside the generated microstructure samples, material properties derived from analytical approximations were used as labels.

Li et al. [110] developed a model to predict the effective modulus of shale, a complex and heterogeneous composite composed of various mineral constituents. The model was trained using Scanning Electron Microscope (SEM) images, which were utilized to generate a large set of stochastic mesoscale samples using a stochastic reconstruction technique. To compute the values of the effective modulus, FEA was used as the basis for the dataset. A CNN was then trained on 10,000 of these generated samples using a standard desktop computer with an Intel i7-8700 central processing unit. The training process required approximately 43 min and included 100 iterations. This showed that the proposed model was more efficient than other ML or DL methods. The model achieved an average prediction error of just 0.97%, indicating strong potential for accurately estimating the effective modulus of real shale samples. Nonetheless, as all training labels were based on simulated data, some influencing factors may not have been fully captured, suggesting that the accuracy of the model should be validated further.

Bakal Gumus et al. [111] confirmed the effectiveness of ANNs in predicting force and deformation in carbon fiber-reinforced composites and BFRCs. Trained using data obtained from three-point bending tests, the ANN model achieved high prediction accuracy, with a strong regression coefficient within R^2^ ≈ 0.987, an average error of 0.0021, and a maximum error of 0.015. Training errors decreased from 0.08 to 0.0012, with the test error stabilized at 0.0020, indicating high generalization and no overfitting. The ANN model offered lower error rates and higher accuracy than traditional methods. Overall, the study demonstrates that ANNs offer a reliable, rapid, and cost-effective approach for predicting the mechanical behavior of composite materials, highlighting the growing role of AI in materials science and engineering applications.

In composite materials literature, damage is defined as any modification to the material or its geometry that results in a change in its mechanical performance [112]. Reliable and cost-effective damage detection is essential for ensuring long lifespans for composite materials and structures [113]. ANNs have emerged as powerful tools in this domain, capable of identifying, localizing, and quantifying damage by analyzing data using non-destructive evaluation [114] and SHM [115] systems. By learning the complex nonlinear relationships between sensor signals and damage states, ANNs can achieve accurate and rapid detection without solely relying on physics-based models [116].

L. et al. [115] proposed an SHM method for composite structures using ANNs. The approach relies on detecting changes in the correlation between strain measurements in a monitored area, as such changes may indicate the presence or growth of damage. Data were collected from a distributed optical fiber sensor embedded in a composite plate during four-point bending tests. A key advantage of the method is its independence from both the type of applied load and the structural material. Unlike conventional ML-based models, this ANN model requires data only from undamaged (”healthy”) conditions and does not rely on damage pattern datasets or data reduction steps. Therefore, it is more time-saving and computationally economical, making it well-suited for practical SHM applications. Additionally, this method demonstrated higher sensitivity to damage-related changes in strain correlations, approximately 200 µε greater than those detected by traditional strain mapping, and was able to detect subtle anomalies that might not raise immediate structural concerns.

Feedforward neural network models are a class of ANNs in which information flows in one direction, from the input layer to the output layer, without any feedback loops, recurrent connections or cycles. They have been employed in conjunction with acoustic-emission techniques to detect damage and enable real-time SHM of composites. In these approaches, acoustic-emission parameters serve as inputs, whereas outputs may include failure modes (classification) [113] or damage tolerance levels (regression) [117]. More recently, models such as CNNs have gained prominence for damage detection and identification tasks [118]. By utilizing image data as input, CNNs have demonstrated improved capability in identifying localized damage features, such as delamination [119]. Furthermore, CNN-based multiscale modeling approaches [120] are being developed to enable end-to-end damage prediction. These models integrate in-process monitoring data that capture microstructures and initial defects, which are obtained through imaging during manufacturing, as input for predicting the performance of the final composite. This strategy supports real-time process intervention, ultimately leading to enhanced quality and performance of composite materials and structures.

Predicting composite material properties under specific production and processing conditions is challenging because of the nonlinear relationship between the input parameters and the resulting material characteristics. To address this, Alcaraz et al. [121] developed a supervised feedforward ANN model to predict and optimize key properties of polyvinyl chloride composites, including tensile strength, ductility, and density, based on the weight percentages of different components, enabling the identification of optimal compositions for achieving specific target properties. Various training algorithms, activation functions, and ANN architectures were examined to capture the complex input–output relationships.

ANNs have also proven to be effective tools for optimizing the fabrication of composite materials, particularly in scenarios involving multiple interacting parameters such as fiber content, curing temperature, layer orientation, printing speed, and infill density. By learning from experimental or simulated datasets, ANNs can model the complex nonlinear relationships between processing conditions and resulting material properties, enabling accurate prediction and process optimization without extensive physical testing [122]. For example, L. Natrayan et al. [123] optimized the manufacturing of pineapple leaf and nano-SiO_2_-based epoxy natural nanocomposites using an ANN model and the hand layup method. The model uncovered the relationships among fiber content, fiber length, alkaline treatment, and nano-silica percentage to predict the bending, tension, and impact-resistance behavior of the fabricated composites. The network structure is depicted in Figure 5.

To optimize drilling parameters for woven glass fiber-reinforced epoxy composites, Abd-Elwahed et al. [124] modeled and optimized spindle speed and feed rate across different laminate thicknesses, with torque and delamination as key performance indicators. They employed response surface methodology (RSM) and ANNs to investigate the effect of these factors on drilling results and their interactions. A PSO algorithm was added to the training process to improve the accuracy of ANN predictions. Predictions from both the RSM and ANN-PSO models were consistent with the experimental results, indicating that they are effective in predicting optimal drilling conditions.

The incorporation of nanofillers into composites has gained considerable research attention owing to enhanced properties, including improved adhesion, stiffness, and overall performance. Natrayan. et al. [125] examined the impact of incorporating varying proportions (0%, 3%, 6%, and 9%) and particle sizes (3, 6, 18, and 25 µm) of graphene into hemp fiber mats of distinct thicknesses (150, 200, 250, and 300 gsm) within epoxy-based nanocomposites. The nanofillers were evenly distributed using high-speed mechanical stirring. The researchers subsequently evaluated several mechanical properties, such as bending or flexural strength and interlaminar shear strength (ILSS). Results showed that the composites composed of 300 g per square meter of hemp and 6 wt.% graphene exhibited the optimal mechanical improvements. An ANN model was developed to predict the mechanical properties of the composites, achieving a strong correlation with the experimental data (R = 0.9724 using the Levenberg–Marquardt algorithm). The prediction errors were less than 3% for bending strength and 4% for ILSS, aligning well with the experimental results and Taguchi L16 matrix outcomes.

Another important domain in which ANNs have demonstrated themselves as powerful and potential tools is the prediction of the mechanical behavior of composite materials subjected to diverse loading conditions, including tension, compression, bending, and shear. These data-driven models are capable of capturing the complex nonlinear relationships between material properties, loading parameters, and structural responses without requiring explicit constitutive equations [19]. Usman et al. proposed a hybrid framework combining crystal plasticity and an ANN model to model the stress–strain behavior and texture evolution in single and polycrystalline AA6063-T6 under tension and shear loads. The key objective was to substantially reduce computational time compared to that required by the traditional crystal plasticity finite element method (CPFEM), without compromising accuracy. Unlike conventional approaches that rely on complex hardening models or large datasets composed of electron backscatter diffraction images, the ANN model employed in their study effectively replicated CPFEM results with minimal error. The ANN model achieved accurate predictions for both stress–strain curves and texture evolution, including pole figure trends and intensities. Notably, it maintained strong predictive performance even beyond the range of the original training data and under non-proportional loading conditions, such as tension followed by shear and vice versa [126].

Fetecau, C. et al. [127] developed ANN models to predict the mechanical properties of 3D-printed polyamide 12 (PA12) and its composites reinforced with carbon fiber (CF) and glass fiber (GF). Using fused filament fabrication and a full factorial design of experiments (DoE), the study examined the influence of printing orientation and infill density on tensile behavior. Mechanical testing results were analyzed using two-way analysis of variance, and regression models were developed for predicting Young’s modulus and tensile strength. Their study demonstrates the potential of integrating DoE with ANN modeling for the reliable prediction of 3D-printed composite performance, enhancing the scope of ANNs in structural applications.

Sahib et al. [128] investigated the use of ANNs in modeling composite sandwich structures. Their study began by solving analytical equations for three-point bending scenarios, followed by generating a training dataset using Monte Carlo simulation. This dataset included various configurations of face sheet laminates, materials, core types, and loading conditions. They developed an ANN model with three hidden layers, each containing 18 neurons and trained using Bayesian regularization backpropagation, which showed high accuracy in predicting the flexural behavior of the structures. The predictions of the model were validated against finite element method simulations and experimental tests using hybrid face sheets, with results showing strong agreement. The study establishes a comprehensive framework for applying ANNs in structural analysis, combining data generation, model training, and validation. It bridges the gap between computational modeling and experimental validation, demonstrating the potential of ANNs as a reliable alternative to traditional methods in structural engineering. This framework can serve as a reference for future research and supports the broader adoption of ML models in the field. The composite sandwich structure is illustrated in Figure 6.

Several studies have investigated the use of hybrid machine learning techniques, particularly the integration of ANNs with other ML methods, to analyze diverse structural configurations and loading conditions. For example, Lefik et al. [130] developed an ANN to predict the response of a two-phase homogenized composite (matrix and fiber) using data from elastic-plastic model simulations, while Yang et al. [131] employed an ANN to predict the T-joint strength of marine sandwich structures, using numerically simulated failure modes as training data. The predictions of the model aligned well with the simulation results. Yong et al. [129] compared three models, namely an adaptive neuro-fuzzy inference system (ANFIS), an ANN, and a simple LR (SLR), for predicting the performance of sandwich structures under axial compression. The ANFIS outperformed the other models, followed by the ANN and then the SLR. Similarly, Akbari et al. [132] integrated a genetic algorithm with an ANN to enhance the tensile strength and hardness of aluminum matrix composites reinforced with particles.

ANN models were also employed to predict the average bending stress and flexural modulus of 3D-printed carbon fiber-reinforced composites across different fiber concentrations. Nawafleh et al. [133] emphasized the critical role of selecting an optimal ANN architecture to ensure accurate and dependable predictions. Even with limited experimental data, the ANN models employed in their study demonstrated a strong predictive performance, closely aligning with the experimental outcomes. Accuracy was assessed using MAPE and CC. The optimum MAPE values obtained for Young’s modulus and bending stress were 8.26% and 6.71%, respectively. These findings demonstrate the effectiveness of ANNs in forecasting the mechanical behavior of composite materials, highlighting their potential as a valuable tool for evaluating the performance of additively manufactured systems. The proposed ANN approach also lays the groundwork for future research on AM-fabricated thermoset composites reinforced with various materials. Table 2 provides a focused comparison of two widely used supervised learning baselines in the composite’s literature: ANN and RF. The percentages reported in Table 2 reflect the distribution only within this section-specific subset of included studies that explicitly employed ANN- or RF-based models as the primary predictive model, rather than across the full corpus of papers considered in this review. Studies that relied primarily on other model families, like linear regression, support vector machines, and Gaussian process models, are discussed in the relevant sections but are intentionally not counted to keep the ANN-RF comparison clear and interpretable.

### 3.2. Unsupervised Learning

This section reviews notable unsupervised learning techniques that have been applied in composite materials research, with a particular focus on PCA for dimensionality reduction, as well as clustering methods such as K-means, fuzzy C-means (FCM), and Gaussian Mixture Models (GMMs), highlighting their roles in pattern recognition, feature extraction, and data interpretation.

Unsupervised learning models operate on datasets lacking explicitly labeled dependent or response variables. In this paradigm, the algorithm autonomously identifies patterns, structures, or relationships within the data without the guidance of predefined outcomes. The principal objective of unsupervised learning is to uncover the inherent structure of the dataset and to facilitate the generation of new hypotheses. Unlike supervised learning, it does not aim to validate existing hypotheses or construct predictive or classification models based on known target variables [134]. In recent years, the integration of AI into the field of composite materials research has shown considerable promise, particularly in the area of SHM and damage diagnosis. Among the various AI techniques, unsupervised learning is gaining increasing attention owing to its ability to uncover hidden patterns in unlabeled data, which is prevalent in experimental materials science, particularly when handling large volumes of AE, ultrasonic, or thermal imaging data [135].

#### 3.2.1. Principal Component Analysis (PCA)

PCA identifies the most significant components within complex datasets by examining correlations among variables [136,137]. It reduces data dimensionality by projecting highly correlated variables onto a smaller set of orthogonal principal components, thereby retaining the most relevant variance while minimizing information loss, as described by Jolliffe et al. [138]. This transformation yields a more concise and interpretable representation of the data, which is particularly valuable for composite systems characterized by numerous interacting factors, such as fiber orientation, volume fraction, matrix type, filler loading, and mechanical responses (e.g., tensile strength, flexural modulus).

In practice, PCA has been thoroughly applied for preprocessing and feature extraction in composite materials studies. For instance, Oskouei et al. [139] combined PCA with fuzzy C-means clustering to analyze AE data in glass/polyester composites. The proposed pipeline effectively enabled clear differentiation among failure modes such as matrix cracking, delamination, and fiber breakage by clustering AE signals in alignment with actual corresponding damage mechanisms. Additionally, Younes et al. [140] investigated the application of PCA to extract dominant structural and operational features in palladium-based composite membranes, demonstrating its effectiveness in simplifying large multivariate datasets and enhancing interpretability in membrane-based energy systems.

#### 3.2.2. Clustering

Clustering algorithms have emerged as powerful machine learning tools capable of effectively analyzing the vast volumes of data generated by modern applications. Their primary objective is to partition data into groups (clusters) such that items within the same cluster exhibit high similarity based on specific distance or similarity metrics. Although extensive research exists on clustering techniques and their applications across domains, practitioners often face challenges due to inconsistencies in the definition of algorithmic properties and the absence of a standardized classification framework. To overcome these limitations, this section presents a comprehensive overview of clustering concepts and algorithms, encompassing both theoretical categorization and empirical evaluations. A structured classification framework was theoretically developed based on key properties identified in prior literature. A series of large-scale empirical experiments was conducted to benchmark representative algorithms from various categories using diverse real-world big datasets. Performance was assessed in terms of internal and external validity indices, stability measures, runtime efficiency, and scalability. The study further identified the clustering algorithms most suitable for big data environments based on the empirical results [141].

In composite materials research, clustering methods are extensively employed to categorize similar materials, detect anomalies, and reveal underlying structures and patterns in experimental or simulated datasets. For instance, clustering has been applied to classify composites based on fabrication conditions, thermal behavior, or failure modes. When handling large-scale data generated from high-throughput experiments or multiscale simulations, clustering helps categorize material variants with similar performance profiles. Additionally, it aids in understanding process–structure–property relationships by identifying natural groupings among different material formulations. Such insights are invaluable in accelerating material discovery and optimizing manufacturing parameters for targeted performance [9].

In the broader framework of unsupervised learning for composite materials, clustering algorithms serve as essential tools for uncovering hidden structures within unlabeled high-dimensional datasets. These methods enable researchers to group data points based on intrinsic similarities, facilitating the identification of material behavior patterns without the need for predefined class labels [20].

The following subsections explore the theoretical foundations, methodological characteristics, and practical applications of each algorithm, emphasizing their role in analyzing AE signals, thermal profiles, and other multivariate datasets in the context of SHM and damage characterization.

##### K-Means

K-means is one of the most straightforward and computationally efficient partitioning clustering algorithms and is widely adopted in composite materials research owing to its simplicity and scalability. The algorithm partitions a dataset into k clusters by minimizing the within-cluster sum of squared distances, typically by iteratively reassigning data points to the nearest centroid and then recalculating the centroid positions until convergence is reached [142]. Although K-means assumes spherical cluster shapes and equal distribution sizes, it remains effective in many practical applications, particularly when combined with dimensionality reduction techniques such as PCA.

In the context of composite materials, K-means has been extensively applied to categorize data based on microstructural attributes, processing parameters, and performance metrics such as tensile strength and fatigue life. This supports pattern recognition, noise filtering, and dimensionality reduction in high-dimensional experimental datasets, enabling researchers to extract dominant trends related to fiber orientations, matrix compositions, and filler distributions [143].

For instance, Zhou et al. [144] employed a hybrid PCA and K-means approach to classify AE features generated during tensile tests on polymer matrix composites. This methodology facilitated automated identification of multiple failure mechanisms, such as matrix cracking and delamination, with over 92% accuracy, thereby reducing reliance on expert interpretation. Similarly, Oskouei et al. [139] combined K-means with self-organizing maps to cluster AE data from glass/polyester composites, successfully distinguishing between different damage types such as matrix cracking and fiber fracture.

In another study, Kurita et al. [145] used K-means to cluster tensile response data from CFRPs, revealing latent performance patterns associated with structural configurations and loading conditions. Additionally, Qiao et al. [146] integrated the K-means algorithm with wavelet packet energy analysis to categorize AE signals acquired from CFRP bending tests into four distinct failure classes, enhancing damage diagnosis accuracy. Özaslan et al. [147] employed PCA and K-means clustering of AE signals to identify damage mechanisms in open-hole composite laminates.

Notably, a preliminary study conducted by Smolnicki et al. [148] examined the clustering of AE data for hybrid fiber-metal laminates using ML approaches. The dataset was clustered into three groups for end-notch flexure tests and into five groups for double cantilever beam tests using K-means, FCM, and spectral clustering. This study demonstrated that ML-assisted clustering of AE data can successfully distinguish damage mechanisms in composites.

##### Fuzzy C-Means (FCM)

FCM, an extension of K-means, introduces soft clustering by assigning a membership value for each data point in a cluster, reflecting the degree to which a point belongs to the cluster. The algorithm minimizes a weighted objective function in which the degree of membership affects the contribution of each data point to the computation of the cluster centroid. FCM is particularly advantageous in composite material analysis when gradual transitions exist between property zones, such as between stiff and flexible regions in functionally graded composites or in hybrid nanocomposites with ambiguous phase boundaries. By capturing the inherent fuzziness in experimental data, such as variability in reinforcement dispersion, FCM provides nuanced insights that hard clustering methods may overlook [9,149].

Fotouhi et al. [150] investigated damage characterization in woven and unidirectional glass/epoxy composites under quasi-static three-point bending tests using AE monitoring, wavelet packet transform, and FCM clustering. The bending tests simulated push-out delamination forces that occur when the composites are drilled without a backup plate. AE signals were decomposed into frequency components using wavelet packet transform. Additionally, the dominant frequency bands were correlated with specific failure modes, such as matrix cracking, fiber and matrix debonding, and fiber breakage, and these correlations were validated through SEM observations. Clustering based on peak amplitude, average frequency, and counts revealed membership distributions consistent with wavelet energy patterns, with notable differences between the two layups. This integrated AE wavelet clustering framework demonstrated high effectiveness as a non-destructive tool for identifying and distinguishing damage mechanisms in composite materials.

Yousefi et al. [151] employed FCM clustering to analyze AE signals generated during low-velocity impact tests on basalt fiber-reinforced polymer laminates. The approach aimed to distinguish among different damage mechanisms by grouping similar damage signals based on their characteristic features. The soft clustering capability of FCM allowed the assignment of membership degrees to multiple clusters, capturing overlaps between damage modes and reflecting the inherent variability in composite failure behavior. The clustering results were validated using post-impact visual inspections and cross-referenced with impact energy levels, demonstrating the effectiveness of FCM as a non-destructive technique for damage identification in impact-loaded composites.

Wang et al. [152] applied FCM clustering integrated with random sequential addition simulation and a custom-developed cellular automata model to quantitatively evaluate the spatial dispersion of carbon fibers in cementitious composites. Backscattered electron micrographs were analyzed using nearest neighbor analysis to obtain dispersion coefficients, which ranged between 0.44 and 0.48. Mechanical and chemical mixing techniques were compared, revealing that up to 6.5 min of mechanically controlled mixing produced a well-distributed conductive network. A negative correlation was observed between dispersion coefficient and electrical resistivity, following a second-order power law. Although microscale fiber arrangement had minimal impact on compressive and flexural strength, it markedly influenced electrical performance, with inconsistencies noted in the chemical mixing system.

##### Gaussian Mixture Models (GMMs)

GMMs provide a probabilistic approach to clustering by modeling the dataset as a mixture of multiple Gaussian distributions, each representing a cluster. Unlike K-means and FCM, GMMs not only provide soft membership but also accommodate elliptical and non-spherical cluster shapes through their covariance structure. Parameters of the distributions (mean, variance, mixing coefficients) are learned using the expectation-maximization algorithm [149].

In composite materials research, GMMs have been used to model the distributions of material properties influenced by process variations or inherent heterogeneity, particularly in particle-reinforced composites and nanocomposites. The probabilistic framework also allows for uncertainty quantification in property predictions, which is essential for designing composites with robust performance under uncertain conditions [9,153].

Wang et al. [154] employed a GMM combined with K-means initialization and the expectation-maximization algorithm to identify structural damage from multi-mode Lamb wave responses. Signal features corresponding to different damage modes were modeled as mixtures of Gaussian probability functions, each defined by a weight, mean vector, and covariance matrix. Baseline GMM parameters were established from the undamaged structural state and used to compare likelihood values for unknown damage cases. The approach effectively distinguished damage types based on maximum likelihood estimation, leveraging the capability of GMMs to model mixed-density distributions with an ellipsoidal classification structure.

### 3.3. Reinforcement Learning (RL)

RL is a prominent ML paradigm that focuses on optimal decision-making through agent–environment interactions. An RL agent learns to select actions that maximize cumulative rewards or achieve predefined objectives by evaluating the quality of different behaviors, rather than following explicitly provided action sequences as in supervised learning. Owing to its reduced reliance on pre-labeled datasets and design flexibility, RL is particularly well-suited for complex and dynamic decision-making scenarios [155]. In composite materials research, RL frames design and processing as a sequential control problem, where states (e.g., temperature/cure history), actions (set-points, dwells), and rewards are optimized under predefined safety constraints. While early applications demonstrated gains in curing and design policies, their success depends on simulation fidelity, appropriate reward formulations, and careful off-policy evaluation to avoid non-transferable policies [86,156,157].

Gongora et al. [156] employed an RL framework to design composite microstructures with a target effective Young’s modulus. The RL agent interacted with a finite element simulation environment, sequentially modifying material distributions to maximize the probability of achieving the desired mechanical property. By leveraging policy optimization and reward shaping, the approach efficiently explored the vast design space, achieving over 90% success in meeting target modulus values while sampling less than 3% of all possible configurations. This methodology demonstrates the capacity of RL to autonomously navigate complex high-dimensional design problems in composite materials research, offering a data-efficient alternative to exhaustive search and enabling accelerated discovery of optimized structures for structural applications.

Extending this to multi-material systems, Raghunath et al. [158] proposed an RL-driven framework to optimize carbon fiber/epoxy resin composites by integrating nanotechnology, advanced manufacturing, and simulation techniques. The methodology incorporated over 10,000 material combinations involving carbon nanotubes, graphene, and polymer matrices, targeting specific performance metrics such as tensile strength and thermal conductivity. The Langmuir–Blodgett self-assembly and electrospinning techniques were employed for fabrication, enabling precise control of nanomaterial dispersion within the matrix. Coupling RL with FEA facilitated the iterative optimization of structure, property and performance relationships, resulting in projected improvements of 15 to 25% in mechanical properties and of 10 to 20% in thermal performance. Simulated thermal expansion coefficients consistently ranged between 1.8 and 3.0, demonstrating both stability and reliability. These findings highlight the potential of RL-based designs to advance composite material performance in aerospace and automotive applications.

Building on the foundations of RL, Deep Reinforcement Learning (DRL) extends this paradigm by integrating the decision-making framework of RL with the powerful capabilities of deep neural networks to learn representations. In DRL, an agent interacts with an environment to learn the optimal policies required for maximizing cumulative rewards, whereas deep networks are employed to approximate complex value functions, policies, or environmental models. This combination enables the agent to handle high-dimensional state and action spaces, process unstructured sensory inputs such as images and audio, and autonomously discover effective strategies to perform complex dynamic tasks [159].

Szarski et al. [160] proposed a DRL-based framework to optimize the curing cycle of carbon fiber-reinforced plastic components in liquid molding processes such as vacuum-assisted resin transfer molding. Recognizing the strong influence of heat transfer variables on production rate and cost, the study framed the oven air temperature profile design as an optimal control problem. A DRL agent, trained in simulation using five 1D heat-diffusion models at different locations, learned an adaptive temperature-control policy capable of anticipating the coupled thermal and chemical dynamics of composite curing. The controller employed both heating and cooling actions to minimize cycle time while satisfying soft constraints on peak system temperature within an acceptable limit and avoiding thermal runaway. This adaptive strategy was integrated into a Bayesian optimization loop to adjust tooling geometry, strategically adding or removing the thermal mass to ensure compliance with hard curing constraints and achieve further reductions in total processing time.

### 3.4. Generative AI

Generative AI refers to AI models and algorithms designed to create new and original content, such as images, text, audio, and data, that is similar to existing data. These models learn the underlying patterns and distributions from training datasets, enabling them to generate novel outputs that preserve the style, structure, and coherence of the source data [44]. Generative AI is primarily built on DL, which enables the model to learn complex patterns from large datasets and generate new content that closely resembles the characteristics of the training data.

In composite materials research, generative AI can be applied to create candidate microstructural configurations, simulate property distributions, and propose material compositions tailored to specific mechanical, thermal, or multifunctional performance targets [161]. The following subsections explore several implementations of generative AI models in composite materials research.

#### 3.4.1. Generative Adversarial Networks (GANs)

GANs are a type of generative AI framework composed of two competing neural networks: a generator, which produces synthetic data, and a discriminator, which evaluates the authenticity of the generated data against real samples. Through adversarial training, the generator progressively improves its outputs until they are indistinguishable from real data, as evaluated by the discriminator [44,58]. Conditional GANs (cGANs) build on standard GANs by conditioning both the generator and discriminator by feeding auxiliary context such as labels or descriptions, producing targeted outputs rather than the random outputs produced by ordinary GANs. Similarly, Khan et al. [162] applied conditional GANs (cGANs) to predict stress distributions in fiber-reinforced adhesive composites used in wind turbine blades. Microcomputer tomography scans were used to capture heterogeneous microstructures, and the images were denoised, segmented, and meshed for FEA under various loading and boundary conditions. The cGAN framework was implemented with Pix2Pix and GauGAN architectures that are designed for image-to-image translation tasks. It learned mappings from microstructure and loading conditions to the corresponding stress fields, effectively translating image data into stress predictions. Despite a limited dataset, the models produced accurate results, with GauGAN showing improved detail preservation over Pix2Pix. This approach enables rapid, image-based mechanical characterization of composites, offering potential applications in defect detection, crack propagation analysis, and multiscale modeling in composite materials research.

Brzin et al. [163] employed GANs for the inverse design of soft morphing composite beams. The proposed framework utilized a generator to create candidate beam geometries that could achieve specified deformation patterns under targeted actuation conditions, whereas the discriminator evaluated the feasibility of these designs based on a dataset acquired from finite element simulations. Through iterative adversarial training, the model successfully produced composite configurations that met the desired morphing performance with high precision. These approaches enable an efficient exploration of the design space for adaptive composite structures, minimizing the reliance on costly physical prototyping.

#### 3.4.2. Variational Autoencoders (VAEs)

VAEs are a type of generative AI that learns a compressed latent representation of data through an encoder–decoder architecture, with a probabilistic latent space that enables controlled generation by sampling and manipulating latent variables [159,160,161].

Sun et al. [164] leveraged VAE’s feature extraction and generative capabilities for the inverse design of fiber-reinforced composite layups. The framework employs multiscale one-dimensional convolutions to extract sequence features from stacking configurations and applies constraints to the latent space to handle the non-uniqueness for layups with certain mechanical properties.

Xue et al. [165] adopted a VAE for the optimization of the inverse design of composite mechanical metamaterials. VAE was chosen to compress representative volume element (RVE) images that describe the spatial distribution of base materials into a reduced latent space. The goal is to design composite mechanical metamaterials with prescribed macroscopic elastic moduli, using the VAE-compressed latent space and Bayesian optimization to automatically find the RVE configuration that achieves them. Similarly, Lyu et al. [166] developed a VAE-CDGM (Conditional Deep Generative Model) framework for the inverse design of composite materials, addressing the limitation of low-quality, blurred images and the vast search space. The VAE-CDGM model synergizes the strengths of VAEs and Denoising Diffusion Probabilistic Models (DDPMs), enabling the targeted design of composite materials while maintaining generation quality. Collectively, these studies emphasize the power of VAEs as a strategy to handle inverse design challenges such as non-uniqueness, search-space complexity, and generation fidelity.

To summarize all of the AI paradigms discussed in this section, Table 3 provides an overview of principles, key advantages, limitations, typical data requirements, and applications in composite materials for each AI algorithm [54,58,164]. These applications demonstrate the growing potential of AI models to accelerate innovation in composite engineering.

## 4. Implications for Composite Materials Research

The integration of AI into composite materials research carries substantial implications, fundamentally transforming the ways in which these advanced materials are designed, developed, and applied. This synergistic approach promises a range of benefits while also introducing distinct challenges that will influence and shape the direction of future research in this field.

### 4.1. Benefits of AI in Composite Materials Research

AI offers several significant benefits in composite materials research, particularly in accelerating discovery, reducing experimental burden, improving predictive accuracy, and supporting optimized design and advanced manufacturing. These advantages make AI an increasingly valuable tool for understanding, designing, and optimizing the processing of complex composite material systems.

1. Accelerated Design and Discovery: Artificial intelligence significantly enhances the pace at which novel composite materials are designed and discovered, enabling the rapid identification and optimization of promising material compositions and architectures. By efficiently exploring extensive compositional spaces and optimizing material properties through learned structure and property relationships, AI enables researchers to predict outcomes prior to physical trials, significantly shortening research cycles. This capability includes applications in the design of new structures with multifunctional properties and the realization of exceptional material characteristics [127,161].
2. Reduced Experimental Testing and Costs: AI models, serving as surrogate models, minimize the necessity for extensive and expensive experimental or computational evaluations by accurately predicting the behavior of composites. This reduction in experimental redundancies and material waste not only enhances sustainability but also leads to substantial cost savings in material development efforts [129,130].
3. Enhanced Accuracy and Efficiency: AI techniques have demonstrated promising predictive accuracy in predicting a wide range of composite material properties, such as mechanical, thermal, and tribological characteristics, frequently surpassing traditional methods. By analyzing vast datasets, these techniques enable efficient predictions for desired outcomes in materials research, revealing complex patterns and relationships that are often challenging for conventional processes to uncover [10].
4. Understanding complex systems: AI’s capacity to identify, classify, and correlate features in intricate material systems enhances our comprehension and control of material behaviors. This is essential for addressing nonlinear failure modes in heterogeneous composite materials [167].
5. Optimal Design Strategies: By investigating the design space and generating optimal solutions, AI assists in exploring strategies for creating high-performing composites, offering new physical insights into the development of tougher and stronger materials. This encompasses the design of composites with specific properties and the selection of optimal architectural configurations [141].
6. Integration with Advanced Manufacturing: AI-guided additive manufacturing techniques, including 3D and 4D printing, paired with machine learning algorithms, create new opportunities for producing complex structures with customized properties and responsive behavior. AI enhances the efficiency and processability of these advanced manufacturing methods, particularly when applied to bio composites [74].

### 4.2. Challenges and Limitations

Despite the significant progress achieved through the application of AI and ML in composite materials research, several challenges and limitations remain. These challenges and limitations can affect model accuracy, reliability, interpretability, and practical implementation. Addressing these issues is essential to improve the robustness, scalability, and real-world applicability of AI-driven approaches in the design, characterization, and optimization of composite materials.

1. Data Scarcity and Quality: A major challenge in applying AI techniques in composite materials is the availability of high-quality, well-labeled datasets. Typically, data from experiments are usually small, with no more than a few hundred data points, which are insufficient to train high-capacity models without running into overfitting issues. Models that have insufficient data relative to their representational capacity tend to fit noise and memorize dataset-specific patterns rather than learn the underlying physics, resulting in performance metrics that are transferable to unseen data. Additionally, data heterogeneity, such as data collected through different fabrication routes and under different testing protocols, can vary in feature representation, measurement scale, and labeling convention, compounding the problem. To resolve this, data-sharing platforms and standardized reporting can increase the volume and reproducibility of available data, and multi-fidelity approaches can allow abundant low-fidelity simulation data to supplement scarce high-fidelity experimental measurements [12,15,30,32].
2. Generalizability: Models trained on a particular dataset often perform poorly on unseen conditions, processing parameters, or material formulations, which limit their practical value across composite systems. This is usually caused by overfitting, since with small or heterogeneous training data, a model learns dataset-specific patterns rather than transferable physical relationships, and the gap between training and validation performance widens as the diversity of the data falls. This makes rigorous data validation approaches, such as K-fold cross-validation and held-out test sets, give a more reliable estimate of performance than a single train–test split, and they should be treated as standard when reporting accuracy in composite material studies. Moreover, transfer learning is an established approach to improved generalization under data constraints, since representations learned on a related task or material system can be adapted to the target application. Regularization techniques such as L1/L2 weight penalties, dropout, and early stopping can reduce model complexity, while ensemble methods improve robustness by aggregating predictions across several models. Claims of generalizability are best supported by testing across different material classes, processing routes, and laboratory sources before a model is deployed in an engineering workflow [7].
3. “Black Box” Nature: Many AI models, particularly deep-learning networks, are frequently referred to as “black boxes” because of their intricate internal processes, which make it challenging to comprehend how different factors affect outcomes. To enhance transparency and interpretability, researchers are increasingly investigating explainable methods and hybrid models that integrate AI with traditional modeling techniques [81].
4. Computational Requirements: While AI lowers computational costs compared to physics-based simulations, training complex AI models, particularly deep-learning networks, still necessitates considerable computing power [142].
5. Need for New Skillsets: The rapid advancement of AI requires a shift in the skillsets needed for researchers, highlighting the importance of data science, programming, a strong theoretical foundation in their field, and a clear vision for discovery [8].
6. Integration with Physics: Although AI is a powerful tool, a significant limitation is its lack of inherent ability to explain the fundamental principles of physics that govern material behavior. To address this concern, an emerging approach involves the development of hybrid models that integrate physics constraints, often referred to as “physics-informed AI/ML.” This approach aims to provide a more comprehensive understanding [143].
7. Active learning and data efficiency: With few true measurements and annotations available, active learning and adaptive sampling, like SVM-guided selection, target the most informative experiments or simulations; i.e., report the acquisition policy, initial design, stopping rule, and total budget so results are comparable [81,168].

The implications of AI for composite materials are far-reaching. It is transforming material development from a trial-and-error process to a data-driven, predictive, and optimized approach. Addressing the identified challenges will be crucial for fully realizing the potential of AI in designing the next generation of high-performance and sustainable composite materials.

## 5. Conclusions and Future Directions

### 5.1. Conclusions

AI and ML integration in composite materials research holds a transformative potential, fundamentally changing how composite materials are designed, synthesized, optimized, and analyzed. This comprehensive review highlights the implications of AI across diverse domains of composite materials science, particularly in expediting design and discovery, enhancing property prediction, optimizing manufacturing processes, and improving damage assessment protocols. Traditional methodologies, which are often characterized by protracted experimental procedures and computationally demanding simulations, are increasingly augmented, if not entirely supplanted, by AI-driven approaches. AI models, notably supervised learning algorithms such as ANNs, SVMs, RF, and DT, have demonstrated strong predictive performance across a range of composite properties, including mechanical strength, thermal conductivity, and wear resistance. ANNs have also demonstrated versatility across various tasks, including the optimization of composite designs, the refinement of manufacturing parameters, and the detection and classification of damage in complex structural systems.

While unsupervised learning techniques, such as PCA and clustering, have received comparatively less attention in the existing literature, they nonetheless provide valuable tools for data analysis, dimensionality reduction, and feature identification. Moreover, DL and generative AI models, exemplified by GANs, are extending the frontiers of research by enabling automated feature extraction, multiscale modeling, and the generation of synthetic data to mitigate challenges related to data scarcity. The synergy of AI with optimization algorithms, particularly Evolutionary Algorithms, has also proven pivotal in efficiently identifying optimal composite configurations.

Notwithstanding these advancements, several formidable challenges impede the widespread adoption and full realization of AI’s potential within the realm of composite materials. The scarcity and heterogeneous quality of available datasets pose significant obstacles to the training of robust AI models, underscoring the necessity for advanced data augmentation strategies and enhanced collaborative efforts for data sharing. Furthermore, the “black box” nature inherent in many complex AI models can undermine interpretability and trust, particularly in applications where safety is critical. Consequently, ensuring the generalizability of these models to untested conditions and diverse material systems constitutes an essential focus for ongoing research in this domain.

Moreover, explainable artificial intelligence and rigorous uncertainty quantification remain underexplored in composite material applications, despite their critical importance for building trust, supporting certification, and enabling the safe deployment of AI-driven models in safety-critical engineering systems. Related progress in digital reticular chemistry offers a helpful analogy for where AI-enabled materials development is heading: pairing a structured design space with data infrastructure, computation, experimental automation, and AI to speed up discovery cycles [89]. In this approach, the goal is not only to predict performance, but to connect prediction with an iterative discovery loop where each round of results guides the next most informative experiments. This framing is valuable for composites because many published models remain limited by small or heterogeneous datasets and by weak tests of generalization beyond the original training domain. A closed-loop strategy, supported by active learning and, where feasible, digital twin-style integration of models with experiments, can improve data efficiency while making model-driven decisions more reliable. For this reason, we highlight closed-loop and physics-aware workflows as practical next steps to help translate AI results from demonstrations into engineering-ready tools. Overall, this review outlines how AI is shaping the future of composite material expansion and identifies opportunities for more efficient and intelligent materials engineering.

### 5.2. Future Directions

For future directions in AI-driven materials science, a practical roadmap combines physics-aware models with data from both simulation and experiments, embedded in closed-loop workflows and digital twins. The goal is to shorten iteration cycles, reduce costs, surface failure modes early, and deliver predictions with trustworthy uncertainty estimates for design and certification. In practice, this means standardizing datasets and metrics, using constraint-respecting surrogates, and wiring models into autonomous labs and process controllers so that each experiment makes the next one smarter. Some potential future research directions are presented in the following list.

1. Physics-Informed Artificial Intelligence: A pivotal avenue for advancement lies in the integration of fundamental physics and domain-specific knowledge into artificial intelligence models. This approach, termed “physics-informed AI,” aspires to enhance the reliability, interpretability, and generalizability of predictive models, specifically in forecasting material behavior with increased precision. By addressing the inherent limitations of traditional black box models, this paradigm fosters a deeper understanding of underlying physical principles. Practical bottlenecks include specifying reliable constitutive laws and boundary conditions, representing material interfaces, and balancing competing terms in the training loss. In composite curing, Niaki et al. [169] required separate temperature and cure subnetworks, sequential training, and adaptive loss weights to address instability and interface behavior. For damage prediction, enforcing an incomplete constitutive law can retain systematic error despite a small physics residual. Priorities should therefore include checking physical assumptions and validating predictions across relevant layups, geometries, and loading conditions.
2. Hybrid Modeling Frameworks: The synergistic combination of AI/ML with conventional computational techniques, such as finite element analysis (FEA) and molecular dynamics, represents a promising research frontier. These hybrid frameworks leverage the complementary strengths of both methodologies, utilizing AI as efficient surrogate models to expedite computationally intensive simulations while ensuring that foundational physical insights underpin the validation processes. Implementation is limited by inconsistent variables and length scales across constituent models and by the cost of generating suitable coupling data. Surrogate errors must be assessed at the component-response level, particularly when stiffness changes or damage initiates. Following the multi-fidelity example in [15], future studies should report how fidelity levels are linked, how discrepancy is estimated, and whether the full workflow remains beneficial after data generation, calibration, and retraining costs are included.
3. Autonomous Experimentation and Digital Twins: The prospective development of AI-driven robotic systems for semi-autonomous experimentation holds substantial promise for the field. This entails the establishment of closed-loop systems wherein AI algorithms continually learn from experimental outcomes, generate new hypotheses, and design subsequent experimental protocols, thereby accelerating the cycles of discovery and development. Furthermore, the creation of comprehensive digital twins, virtual representations of physical composite materials and structures, will facilitate real-time monitoring, predictive maintenance, and performance optimization throughout the material lifecycle. A functioning twin requires repeated assimilation of measurements into a model of a specific component or process. Kapteyn et al. [170] demonstrated experimental calibration of an unmanned aerial vehicle (UAV) structural twin, while its damage-response scenario used synthetic observations, illustrating the distinction between calibration and operational validation. For composite production, practical obstacles include sparse internal-state measurements, sensor drift, update latency, and integration with existing controllers. A useful next step is a twin for a defined task, such as cure monitoring, with independent process validation, explicit uncertainty thresholds, and a fallback procedure when measurements or predictions become unreliable.
4. Large Language Models and Multimodal AI: The emergence of large language models heralds a transformative future in which AI can effectively process and integrate diverse types of data, including textual, visual, and contextual information, alongside conventional material parameters. This evolution may lead to the emergence of multimodal AI/ML frameworks capable of addressing complex challenges in composite design and damage mechanics. Practical use is constrained by factual errors and by the need to associate each extracted value with its material, units, and test conditions. Polak et al. [171] demonstrated materials-data extraction with explicit verification steps, but errors remained [15,172]. For composites, multimodal training additionally requires consistent links between specimen descriptions, micrographs, manufacturing histories, and measured responses. Domain adaptation can also require substantial computational resources and access to proprietary records. Near-term applications should prioritize source-linked extraction and retrieval from curated records, with expert checks and independent benchmarks before outputs inform material selection or damage assessment.
5. Data Infrastructure and Standardization: To underpin the data-driven paradigms of AI, it is imperative to continue developing extensive, high-quality databases of material properties and to standardize protocols for data collection and sharing across both academic and industrial landscapes. For composites, a proposed minimum record should include matrix and reinforcement identity, fiber volume fraction and architecture or layup, processing history, specimen geometry, conditioning, loading direction and rate, and the test method and version. Raw measurements, replicate counts, uncertainty estimates, and specimen and batch identifiers should accompany reported properties. Simulation records should additionally identify constitutive models, mesh resolution, boundary conditions, solver version, and fidelity level. Shared units, coordinate conventions, and controlled terminology are necessary to prevent incompatible measurements from being pooled. Public databases could be established through a consortium of laboratories and manufacturers that agree on this schema, validate submissions, and maintain versioned benchmark datasets and comparison protocols. The FAIR principles support persistent identifiers, provenance, interoperable metadata, and explicit reuse licenses [159].
6. Addressing Uncertainty Quantification: There is an urgent need for ongoing research focused on the quantification and management of uncertainties that are inherently associated with AI predictions. This effort is crucial to ensuring the robustness and reliability of AI-assisted composite designs, especially in the context of critical engineering applications. Building on Section 4.2, priorities should include calibration benchmarks with independent batches and explicit tests under changes in material, process, and environment. Because calibrated uncertainty can remain unreliable outside the model domain [160], uncertainty estimates should be coupled to applicability checks and predefined decisions, such as acquiring additional measurements or deferring automated acceptance. These checks should be repeated after model updates.

By integrating rigorous data practices, physics-aware modeling, and transparent uncertainty quantification, AI has the potential to move composite materials from promising prototypes to validated and certifiable solutions for high performance and sustainable structures. In the coming years, the most meaningful progress will likely come from studies supported by open, well-annotated datasets, reproducible baselines, and clear reporting of uncertainty.

## Figures and Tables

**Figure 1:**
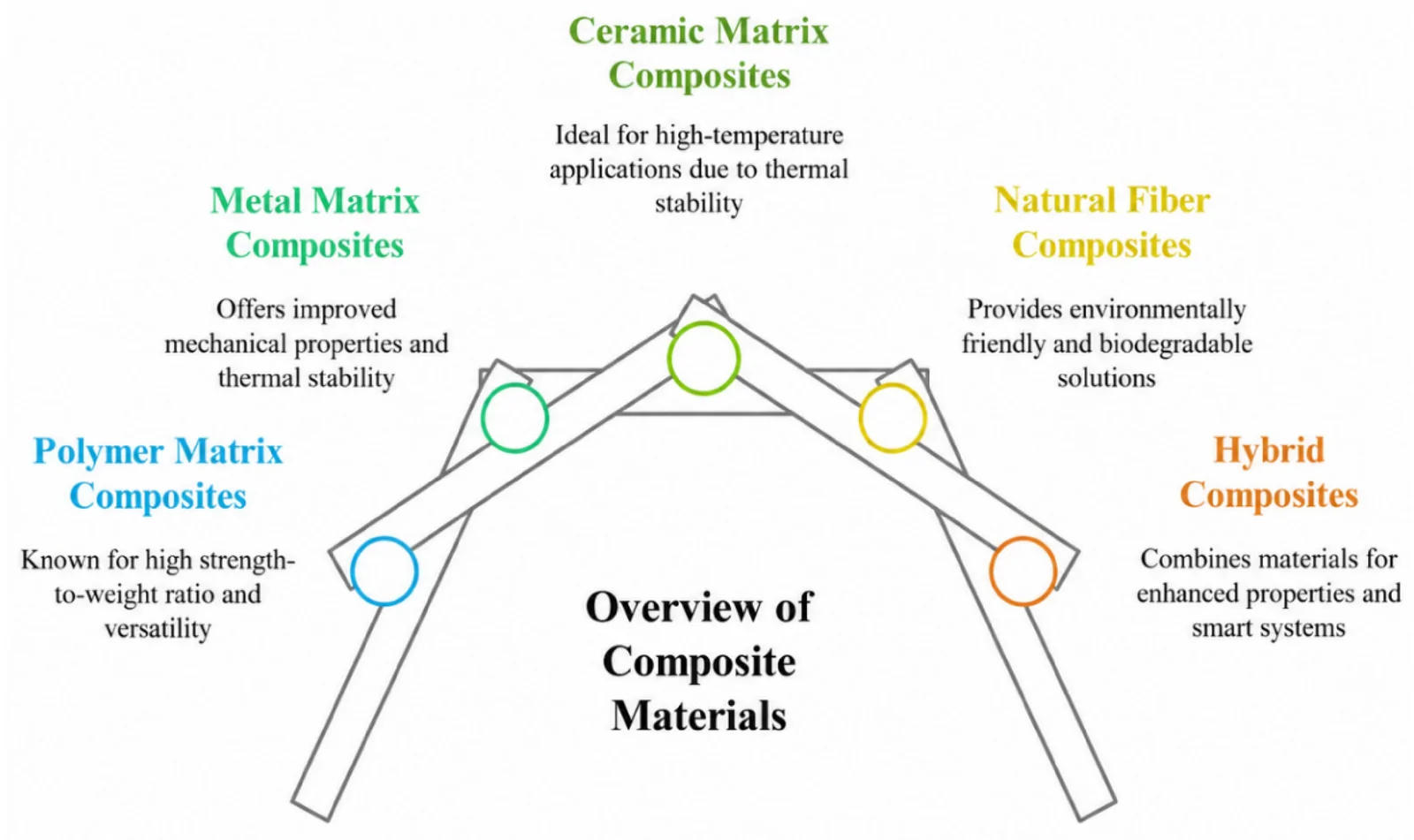
An overview of composite materials.

**Figure 2:**
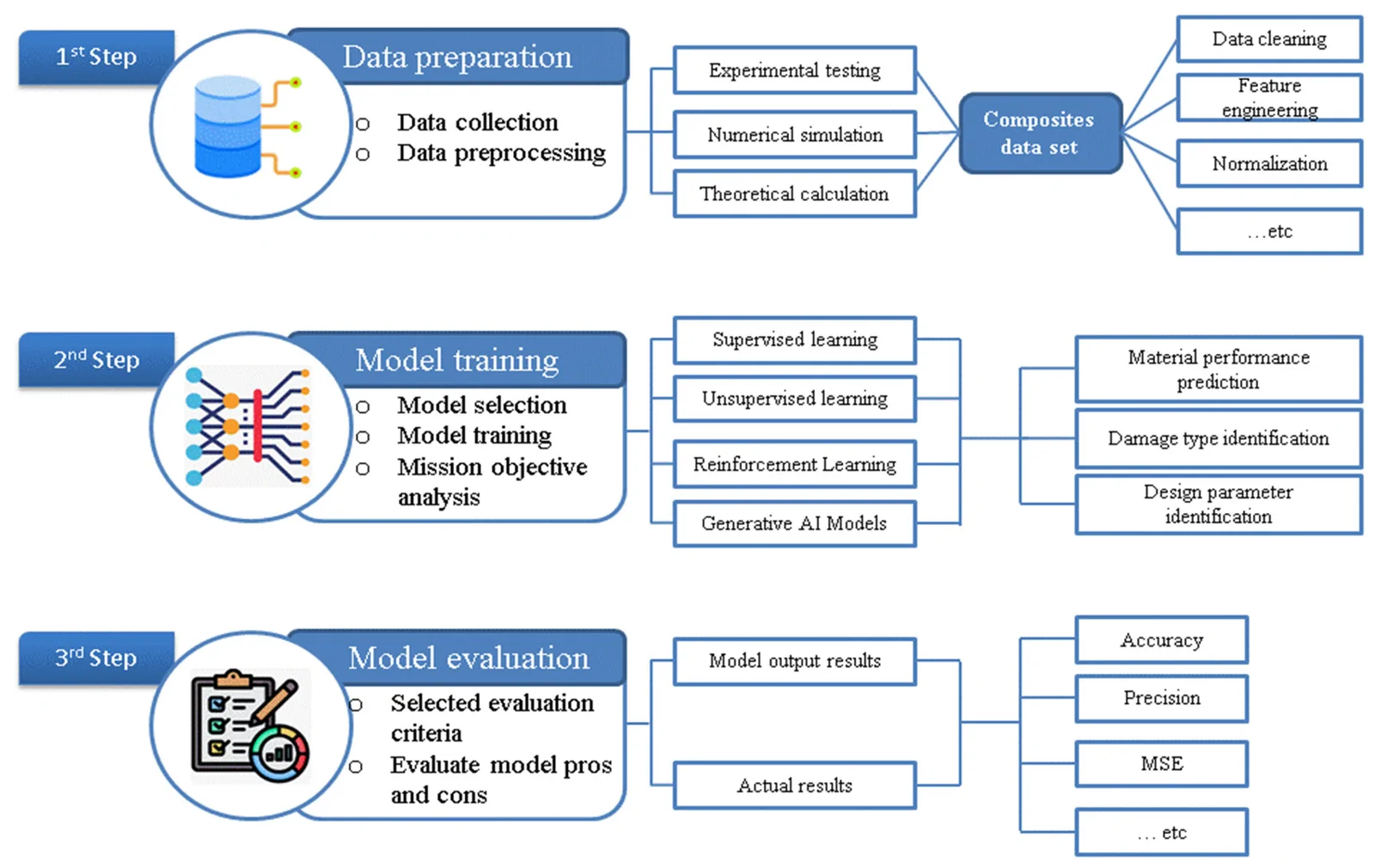
General machine learning workflow showing the main stages of data preparation, model training, and model evaluation.

**Figure 3:**
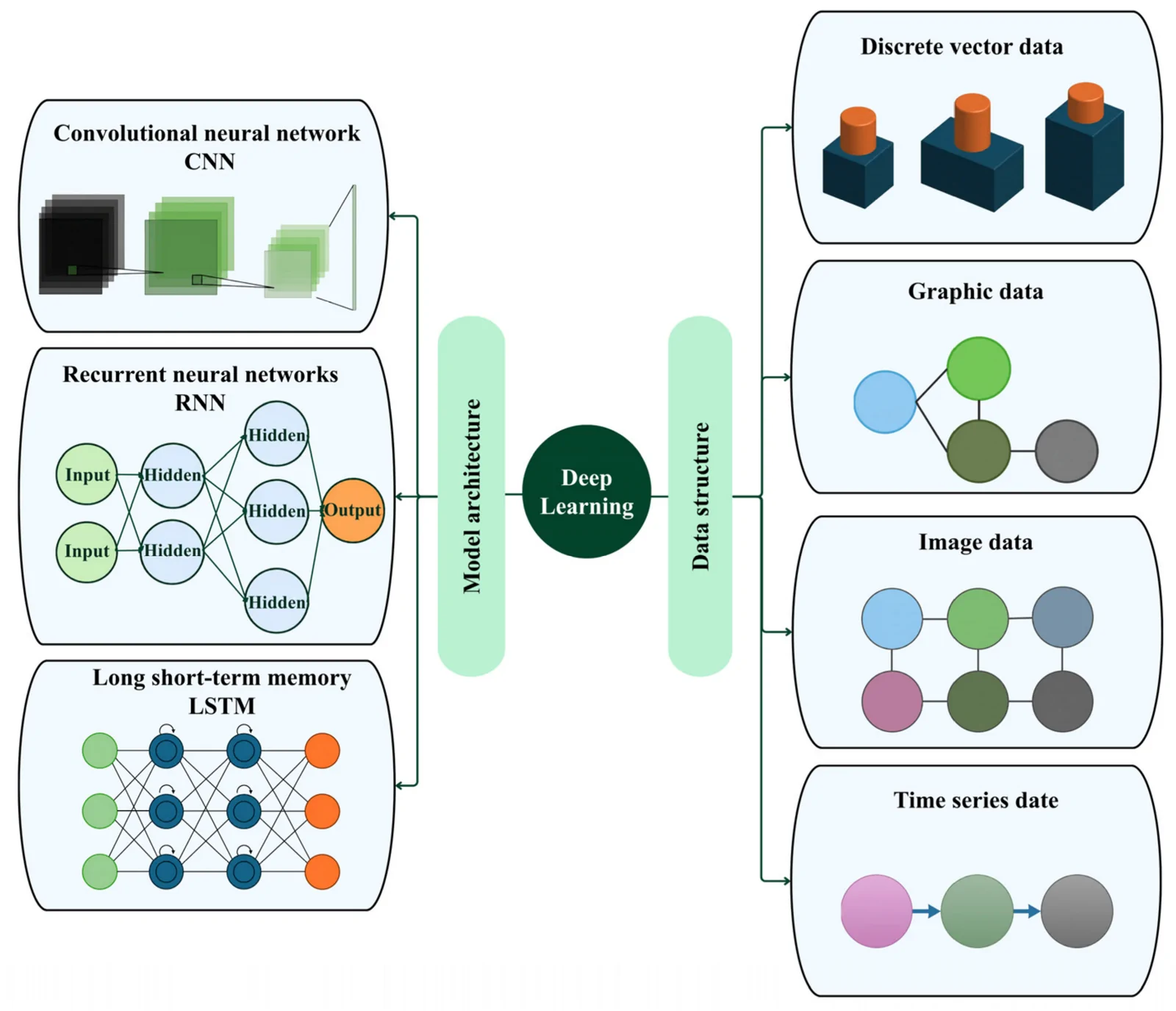
General deep-learning framework showing representative neural network architectures and the main types of data structures that can be processed using deep-learning models.

**Figure 4:**
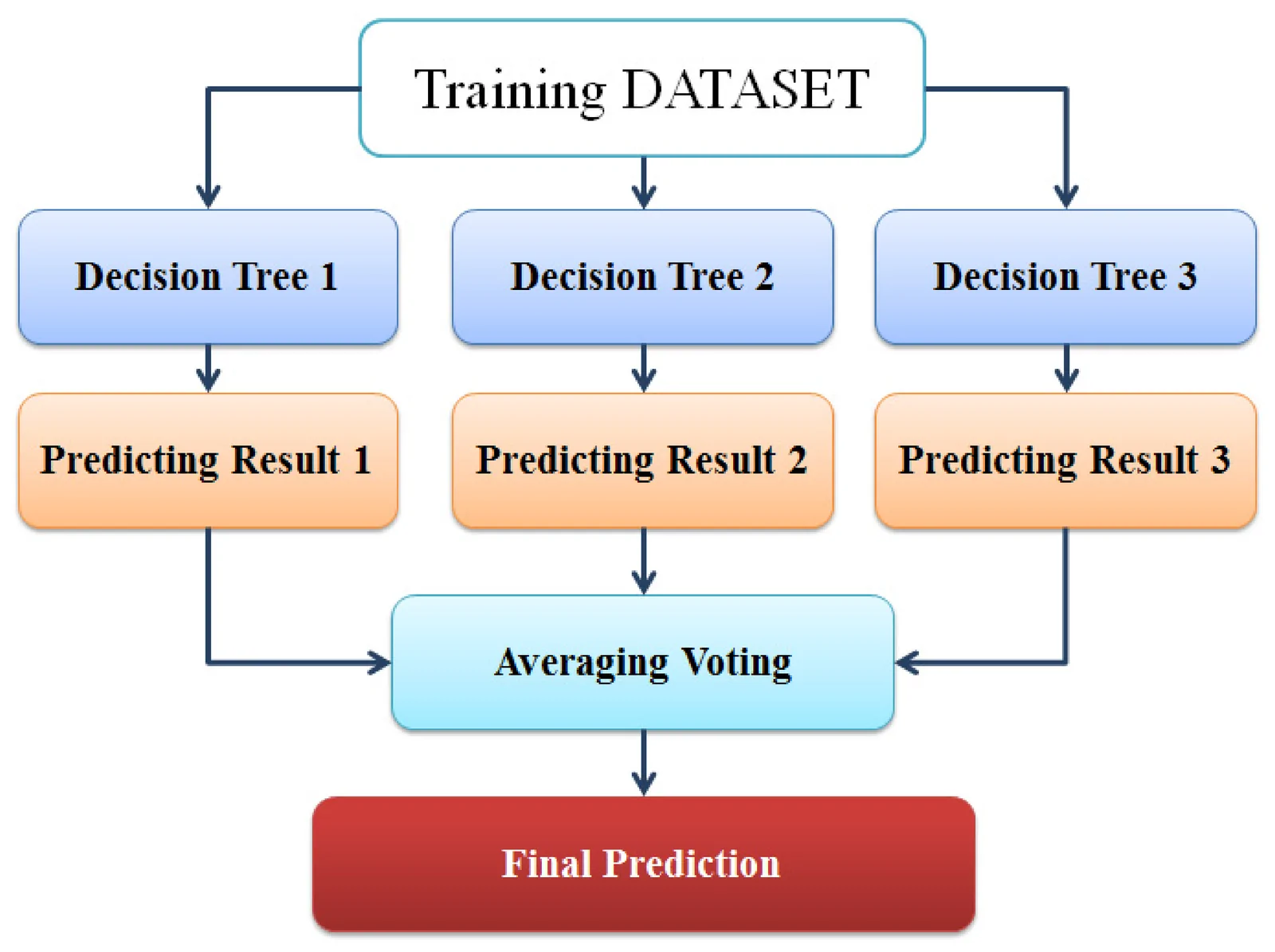
A demonstration of regression using a random forest [98].

**Figure 5:**
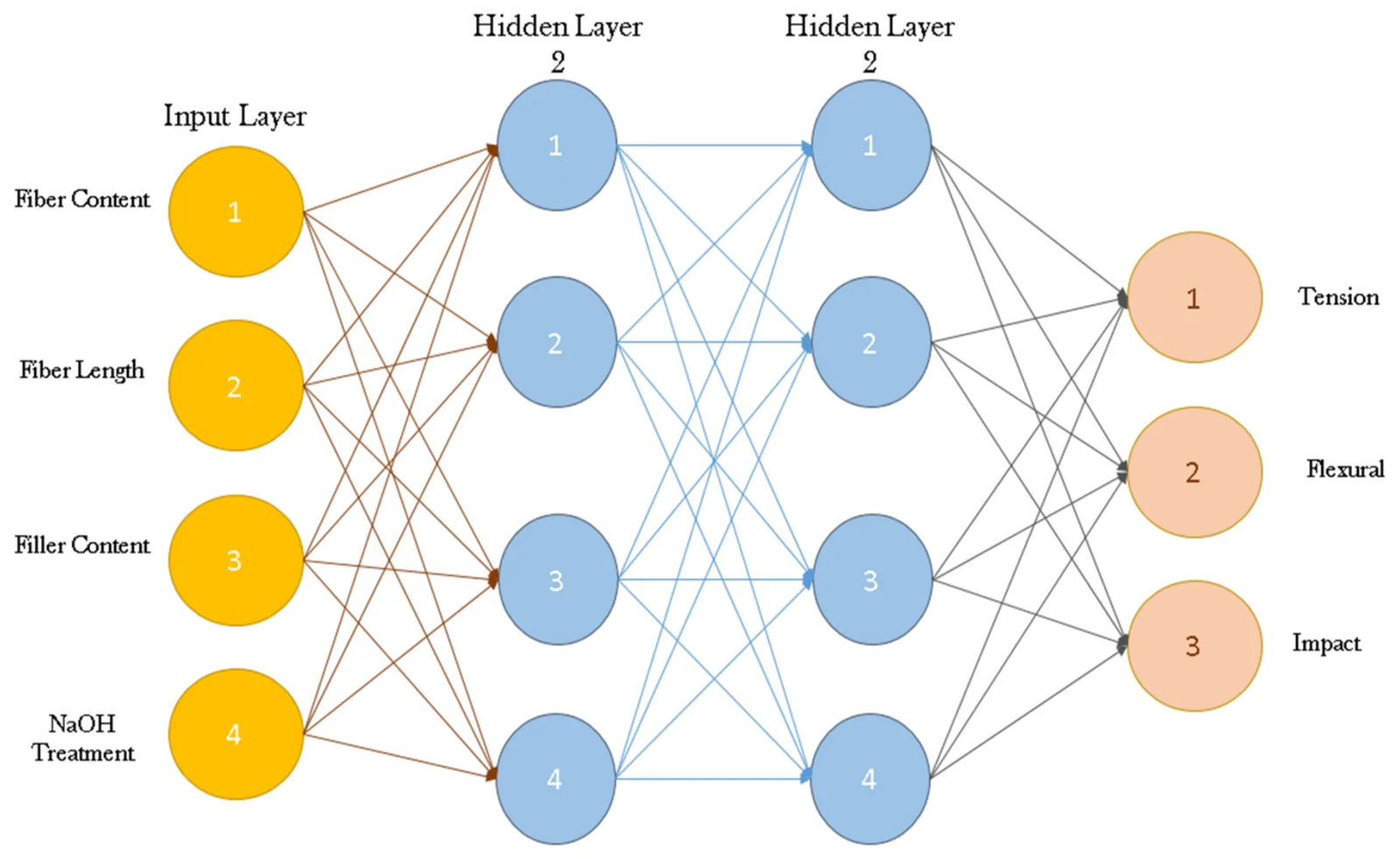
ANN structure for L. Natrayan et al.’s research work [123].

**Figure 6:**
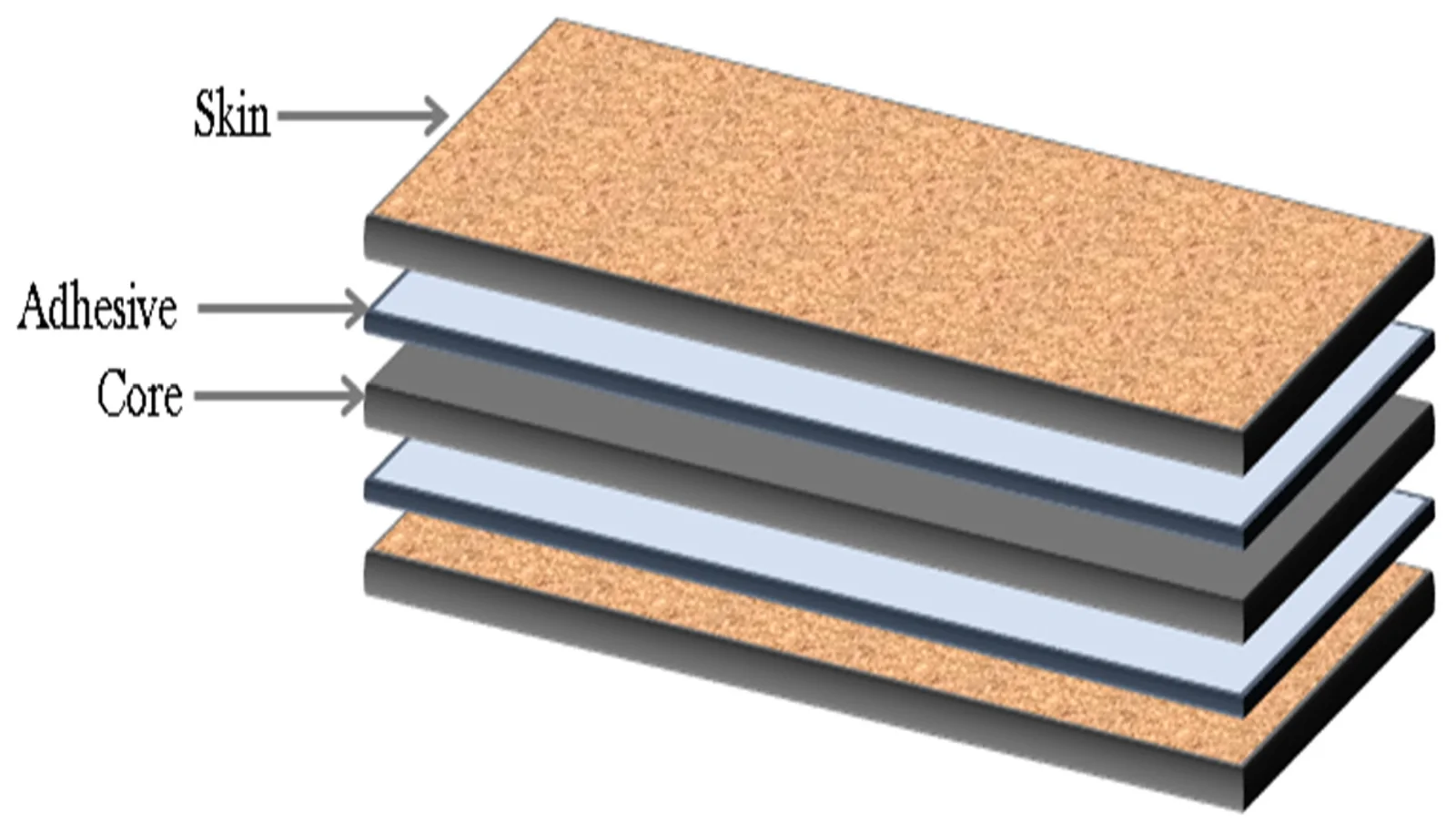
Schematic of a sandwich structure [129].

**Table 1:** Distribution of eligible AI–composite studies across AI techniques and composite material groups.

Category		Percentage, % (Eligible Studies/Total References)
AI Technique group	Traditional ML	33.3/11.0
	Artificial neural networks (ANN)	22.8/7.6
	Deep learning	15.8/5.2
	Generative/latent-space AI	8.8/2.9
	Unsupervised learning/clustering	10.5/3.5
	Reinforcement learning	5.3/1.7
	Hybrid AI/optimization	3.5/1.2
Composite material groups	General/heterogeneous composites	40.4/13.4
	Polymer/hybrid composites	21.1/7.0
	Fiber-reinforced polymer composites	19.3/6.4
	Structural/architected composites	7.0/2.3
	Metal-matrix composites	5.3/1.7
	Ceramic/cementitious composites	7.0/2.3

**Table 2:** Distribution of ANN- and RF-based studies. Percentages are calculated within the ANN/RF subset only and do not represent the full corpus of 172 reviewed papers.

AI Model	Percentage (%)
Artificial Neural Networks (ANN)	80
Random Forest (RF)	20

**Table 3:** Summary of AI paradigms, including their principles, advantages, limitations, data requirements, and applications in composite materials.

**Composite Material** **Applications**	Microstructure identification from micro-CT; property prediction for polymer systems	FDM surface-roughness optimization; stacking-sequence selection	Bond-strength prediction for FRP rods; AE signal analysis; tensile-strength estimation	Mechanical and thermal property prediction; wear modeling; SHM	Defect and delamination detection from C-scan, thermography, micro-CT	Cure monitoring; fatigue and creep evolution; AE sequence classification	AE signal preprocessing; feature extraction prior to clustering
**Typical Data Requirement**	Small–moderate data; labeled tabular data; feature scaling	Small labeled tabular data; tolerant of unscaled and categorical inputs	Moderate size; labeled tabular data; tolerates missing values	Large labeled data; normalization required	Large images or signal data	Sequential or temporal data	Unlabeled continuous multivariate data; scaling is essential
**Limitations**	Hyperparameter sensitivity; slow on high-dimensional data	Suffers from overfitting and generalization	Computational complexity with high-dimensional data	Requires high-quality data; computationally complex; interpretability (black box)	Requires large labeled sets; computationally costly; limited extrapolation	Training instability; sequential computation limits parallelism	Struggle with nonlinear data and outliers
**Key Advantages**	High performance with limited samples; Handles linear & nonlinear data	Highly interpretable; handles nonlinear data	Reduces overfitting; native feature-importance ranking; robust to noise	Models complex nonlinear multivariate relationships	Learns spatial features without manual engineering; state of the art for imaging	Captures sequential and time-dependent behavior	Minimizes information loss; interpretability of complex datasets.
**Principle**	Constructs hyperplanes to separate classes	Hierarchical partitioning of feature space into branches	Ensemble of Decision Trees using bagging and majority voting/averaging.	Multilayered nodes that learn complex patterns via activation functions and backpropagation	Convolutional feature extraction from gridded data	Recurrent units retaining temporal state	Reduces dimensionality by projecting correlated variables into orthogonal components
**Algorithm**	SVM	DT	RF	ANN/MLP	CNN	RNN/LSTM	PCA
**Paradigm**	Supervised	Supervised	Supervised	Supervised	Supervised (DL)	Supervised (DL)	Unsupervised

## Data Availability

No new data were created or analyzed in this study. Data sharing is not applicable to this article.
